# Proteomics Profiling to Distinguish DOCK8 Deficiency From Atopic Dermatitis

**DOI:** 10.3389/falgy.2021.774902

**Published:** 2021-11-29

**Authors:** Minnie Jacob, Afshan Masood, Zakiya Shinwari, Mai Abdel Jabbar, Hamoud Al-Mousa, Rand Arnaout, Bandar AlSaud, Majed Dasouki, Ayodele A. Alaiya, Anas M. Abdel Rahman

**Affiliations:** ^1^Metabolomics Section, Department of Clinical Genomics, Center for Genomics Medicine, King Faisal Specialist Hospital and Research Centre, Riyadh, Saudi Arabia; ^2^Proteomics Resource Unit, Obesity Research Center, College of Medicine, King Saud University, Riyadh, Saudi Arabia; ^3^Proteomics Unit, Stem-Cell and Tissue Re-engineering Program, King Faisal Specialist Hospital and Research Centre, Riyadh, Saudi Arabia; ^4^Section of Pediatric Allergy and Immunology, Department of Pediatrics, King Faisal Specialist Hospital and Research Centre, Riyadh, Saudi Arabia; ^5^Department of Biochemistry and Molecular Medicine, College of Medicine, Alfaisal University, Riyadh, Saudi Arabia; ^6^Department of Chemistry, Memorial University of Newfoundland, St. John's, NL, Canada

**Keywords:** atopic dermatitis, dedicator of cytokinesis (DOCK8), hyper IgE syndrome (HIES), label-free proteomics, biomarker, multiple reaction monitoring

## Abstract

Dedicator of cytokinesis 8 deficiency is an autosomal recessive primary immune deficiency disease belonging to the group of hyperimmunoglobulinemia E syndrome (HIES). The clinical phenotype of dedicator of cytokinesis 8 (DOCK8) deficiency, characterized by allergic manifestations, increased infections, and increased IgE levels, overlaps with the clinical presentation of atopic dermatitis (AD). Despite the identification of metabolomics and cytokine biomarkers, distinguishing between the two conditions remains clinically challenging. The present study used a label-free untargeted proteomics approach using liquid-chromatography mass spectrometry with network pathway analysis to identify the differentially regulated serum proteins and the associated metabolic pathways altered between the groups. Serum samples from DOCK8 (*n* = 10), AD (*n* = 9) patients and healthy control (Ctrl) groups (*n* = 5) were analyzed. Based on the proteomics profile, the PLS-DA score plot between the three groups showed a clear group separation and sample clustering (*R*2 = 0.957, *Q*2 = 0.732). Significantly differentially abundant proteins (*p* < 0.05, FC cut off 2) were identified between DOCK8-deficient and AD groups relative to Ctrl (*n* = 105, and *n* = 109) and between DOCK8-deficient and AD groups (*n* = 85). Venn diagram analysis revealed a differential regulation of 24 distinct proteins from among the 85 between DOCK8-deficient and AD groups, including claspin, haptoglobin-related protein, immunoglobulins, complement proteins, fibulin, and others. Receiver-operating characteristic curve (ROC) analysis identified claspin and haptoglobin-related protein, as potential biomarkers with the highest sensitivity and specificity (AUC = 1), capable of distinguishing between patients with DOCK8 deficiency and AD. Network pathway analysis between DOCK8-deficiency and AD groups revealed that the identified proteins centered around the dysregulation of ERK1/2 signaling pathway. Herein, proteomic profiling of DOCK8-deficiency and AD groups was carried out to determine alterations in the proteomic profiles and identify a panel of the potential proteomics biomarker with possible diagnostic applications. Distinguishing between DOCK8-deficiency and AD will help in the early initiation of treatment and preventing complications.

## Introduction

Dedicator of cytokinesis 8 deficiency is a rare autosomal recessive combined immunodeficiency that forms part of the heterogeneous hyper immunoglobulin E syndrome (HIES). dedicator of cytokinesis 8 (DOCK8) is a guanine nucleotide exchange factor that regulates the activity of cell division cycle 42 (Cdc42) necessary for promoting actin binding, maintaining cytoskeletal integrity, and integrating signals from the cell membrane for appropriate cytoskeletal reorganization (1). Biallelic, homozygous, and heterozygous DOCK8 mutations have been reported with frequent large deletions and point mutations, leading to either loss of function or expression of trace amounts of protein (2). DOCK8 is a cytoskeletal protein, highly expressed in the hematopoietic cells and normal peripheral blood mononuclear cells, especially lymphocytes, neutrophils, and expressed in the placenta, kidney, lung, and pancreas (3). Functionally, DOCK8 plays an important role in cell migration, morphology, adhesion, and growth (4). The characteristic features associated with DOCK8 deficiency include elevated IgE levels, recurrent bacterial and viral infections, atopic dermatitis, mucocutaneous candidiasis, asthma, severe food and environmental antigens allergies, and an increased incidence of malignancy (5, 6). Individuals with DOCK8 mutations exhibit recurrent sinopulmonary infections specific for humoral immunodeficiency and severe viral infections suggestive of T-cell dysfunctions (7). In addition, patients with DOCK8 deficiency characteristically manifest with dermatitis-like skin lesions resembling atopic dermatitis (AD).

Atopic dermatitis or eczema is a prevalent chronic inflammatory skin disease, and specific food allergens and nutrients are closely related to the development and severity of the disease (8). The clinical picture of atopic dermatitis (AD) overlaps with DOCK8 deficiency, but, unlike the latter, patients with AD are mostly susceptible to superficial infections with Staphylococcus aureus, and deep-seated infections rarely occur. Due to overlapping symptoms in these patients, differentiating patients with DOCK8 deficiency from those with AD is critical as treatment modalities in both conditions are substantially different. Topical corticosteroids, systemic antibiotics, and antifungal agents are used for prophylactic and symptomatic treatment in conjunction with topical therapy in AD. At the same time, hematopoietic stem cell transplantation (HSCT) is the preferred definitive therapy for DOCK8 deficiency, which, when initiated early, reduces the high morbidity and mortality associated with it (9).

Previous studies by our group have reported distinct alterations in levels of cytokines and potential metabolomics biomarkers distinguishing patients with DOCK8 deficiency from AD (10, 11). Proteomics has evolved as a promising platform for identifying signature proteomic profiles and their interactions between disease states and controls. Untargeted proteomic profiling can accurately determine the relative abundance of proteins within tissues and offers the opportunity to comprehensively investigate the changes in signaling pathways and biological processes that would otherwise be missed by transcriptome sequencing (12, 13). To date, we could not identify any studies that have characterized the proteome of patients with DOCK8 deficiency. The present study label-free, quantitative proteomics approach was used to identify the differentially regulated proteins in the disease states and compare the proteomes between DOCK8 deficiency and AD (14, 15). Differentially expressed proteins were identified, and their association with the disease phenotype was introduced using network pathways analyses. The findings of the study were validated using liquid chromatography multiple reaction monitoring (MRM) tandem mass spectrometry (LC-MS/MS).

## Materials and Methods

### Chemicals

Analytical solvents, Dithiothreitol (DTT), and Iodoacetamide (IAA) were purchased from Sigma-Aldrich (St. Louis, Missouri, USA) and RapiGest SF from (Waters, UK). Proteins signature peptides were synthesized and obtained from GeneMed Synthesis (San Franciso, CA, USA).

### Characteristics of the Study Population

Through the Allergy and Immunology clinics at KFSHRC, subjects with a genetically confirmed hereditary DOCK8 deficiency and severe AD meeting the Hanifin and Rajka clinical criteria and healthy controls (Ctrl) were consented to participate in this study (16). The study participants comprised of three groups: DOCK8 deficient (*n* = 10) and AD (*n* = 9) patients, and Ctrl (*n* = 5). Patients who received bone marrow transplantation, enrolled in another clinical study, unwilling to provide informed consent, or whose sample amount was not sufficient were excluded from the study. A baseline questionnaire, including clinical symptoms, allergies, and family history, was collected. This study was approved by the Research Ethics Committee at the Office of Research Affairs of King Faisal Specialist Hospital and Research Centre (KFSHRC) (RAC No. 2160015).

### Proteomics Analysis on LC-MSE SynaptG2

The serum samples were depleted, protein concentrations were normalized, and 100 μg of total depleted protein from each sample was subjected to in-solution tryptic digestion as previously described (14). Concisely, proteins were denatured at 80°C for 15 min, reduced in 10-mM Dithiothreitol at 60°C for 30 min, and alkylated in 10-mM Iodoacetamide at room temperature in the dark for 40 min. Proteins were trypsin digested at 37°C for 10–12 h. All samples were diluted with 1% formic acid to a concentration of 1 μg/μl before LC-MS.

Label-free quantitative liquid chromatography coupled to Synapt G2 mass spectrometry (Waters, Manchester, UK) was used to generate expression protein profiles between the sample groups. The instrument settings were optimized, including the detector set up using 2 ng/μl Leucine Enkephalin and an instrument calibrated using [Glu1]-Fibrinopeptide B Standard (GFP) (Waters, UK) (15). All Trizaic Nano source and ionization analyses were performed in the positive ion mobility mode Nano ESI (Waters, Manchester, UK). Data-independent acquisition (MSE)/ion mobility separation was performed, and data were acquired over a range of m/z 50–1300 Da using the Mass Lynx program (version 4.1, SCN833, Waters, Manchester, UK). All samples were analyzed in the same batch, and each sample was in triplicate runs as previously described (15).

The mass spectrometry proteomics data have been deposited to the ProteomeXchange Consortium *via* the PRIDE partner repository with the dataset identifier PXD029052.

### Multiple Reaction Monitoring–Tandem Mass Spectrometry for Validation

A signature peptide per protein from the proteomics profile was selected as described previously and confirmed using SkyLine Software V3 (17, 18). The tryptic digests of patient samples were purified using solid-phase extraction (SPE) (19). The peptides were initially separated by reversed-phase chromatography using Acquity Ultra Performance Liquid Chromatography (UPLC) C18, 1.7-μm and 2.1-×-50-mm columns, 25°C in 10 min, and a flow rate of 0.2 ml/min in a positive ionization mode. The desolvation and source temperatures were set at 250°C and 150°C, respectively. The spraying gas flow rate was 500 L/h, with a sample flow rate of 20 μl/min. The source and cone voltages were set at 1.98 KV and 47 V, respectively. Multiple Reaction Monitoring (MRM) was established using the optimum parameter for each peptide as described previously (19).

### Data Analysis

Samples were analyzed in triplicate runs. Automated data processing and database search were performed using the Uniprot protein sequence database on Progenesis QI for proteomics (QIfP V 3.0.6039) for differential expression analysis (Waters, Manchester, UK).

The raw data were normalized, log transformed, and Pareto scaled, and then, based on a fold-change (FC) criterion of >2 and a false discovery rate (FDR) adjusted *p*-value < 0.05, a univariate analysis (volcano plot) was performed for each binary comparison to identify significantly differentially expressed proteins. Multivariate analysis, partial least square-discriminant analysis (PLS-DA) was performed using MetaboAnalyst V4 (McGill University, QC, Canada) (20). The clustering between group replicates is indicated of technical reproducibility of the sample preparation and analysis. Further analysis, including receiver-operating characteristic (ROC) curves, was constructed using the PLS-DA method. All PLS-DA models were cross-validated (leave-one-out cross validation) and passed permutation tests (100 permutations). The dataset was split into 70:30% for training to testing purposes. *R*2, Q2, and *p*-values for permutation tests are provided in the caption of each PLS-DA score plot.

### Bioinformatics and Pathway Analysis

The functional and biochemical interactions of the proteome profile were networked using Ingenuity Pathway Analysis (IPA) software (QIAGEN, USA). The core analysis was carried out for the different data sets to identify the relationships and pathways relevant to each dataset. The software calculated a score based on the number of molecules in the network.

## Results

### Clinical Characterizations of Patients With DOCK8 Deficiency and AD

The demographic data of the study participants have been described previously (11). The mean age of the DOCK8-deficient and AD cohorts was 13.2 ± 5.9 and 10.8 ± 1.4 years, respectively, and the Ctrl was 23 ± 1.03. The CD4/CD8 ratio in the DOCK8-deficient cohort (2.8 ± 0.99) was double when compared to the AD cohort (1.43 ± 0.14). Eosinophilia was seen in all the patients, albeit the counts were higher in the DOCK8-deficient cohort than AD; high counts in DOCK8 deficiency cannot be used as the only criterion for diagnosis eosinophils can be elevated in many other diseases. At the same time, neutrophil counts were not significant in both cohorts. The mean RBCs and WBCs counts in patients with DOCK8 deficiency were 4.5 ± 0.5 (1012/L) and 10.53 ± 2.3 (109/L), whereas, in patients with AD, they were 5.3 ± 0.16 (1012/L) and 6.74 ± 0.9 (109/L), respectively. The Severity Scoring of Atopic Dermatitis (SCORAD) and the Visual Analog Scale (VAS) pruritus scores were comparable in both cohorts (Table 1). Atopic dermatitis, food allergies, pneumonia, and staphylococcal infections were the most commonly seen clinical presentations in the DOCK8 cohort. In patients with AD, pneumonia or deep-seated staphylococcus infections were not observed. As expected, total IgE levels in both DOCK8-deficient and AD groups were elevated when compared to the Ctrl groups, with patients with DOCK8 deficiency, showing significantly higher serum IgE levels (*p* < 0.05) compared to patients with AD and Ctrl (5–500 KU/L). Splicing mutations were the most common (64%), followed by deletion mutations (27%) and stop codon mutations (9%) in the DOCK8-deficient cohort, as described previously (10).

**Table 1:** A summary of clinical demographics and laboratory readings of patients; the values are represented as the average and standard error of the mean (average ± SEM).

**Diagnosis** **(Average ± SEM)**	**Age** **(Y)**	**Sex** **(M/F)**	**IgE Levels** **(KU/L)**	**RBC** **10^**∧**^12/L**	**WBC** **10^**∧**^9/L**	**Eosinophils** **10^**∧**^9/L**	**CD4/CD8** **Ratio**	**VAS**	**SCORAD**
DOCK8	13.2 ± 5.9	5/5	19817.30 ± 4772.6	4.5 ± 0.57	10.53 ± 2.3	27.29 ± 4.02	2.8 ± 0.99	14.1 ± 0.75	67.26 ± 2.37
Atopic dermatitis	10.8 ± 1.4	8/1	5288.20 ± 1736.3	5.33 ± 0.16	6.74 ± 0.92	11.5 ± 1.3	1.43 ± 0.14	13.6 ± 0.86	63.5 ± 3.702

*F, female; M, male; VAS, visual analog scale; SCORAD, scoring atopic dermatitis*.

### Proteomics Profiling

**Figure 1:**
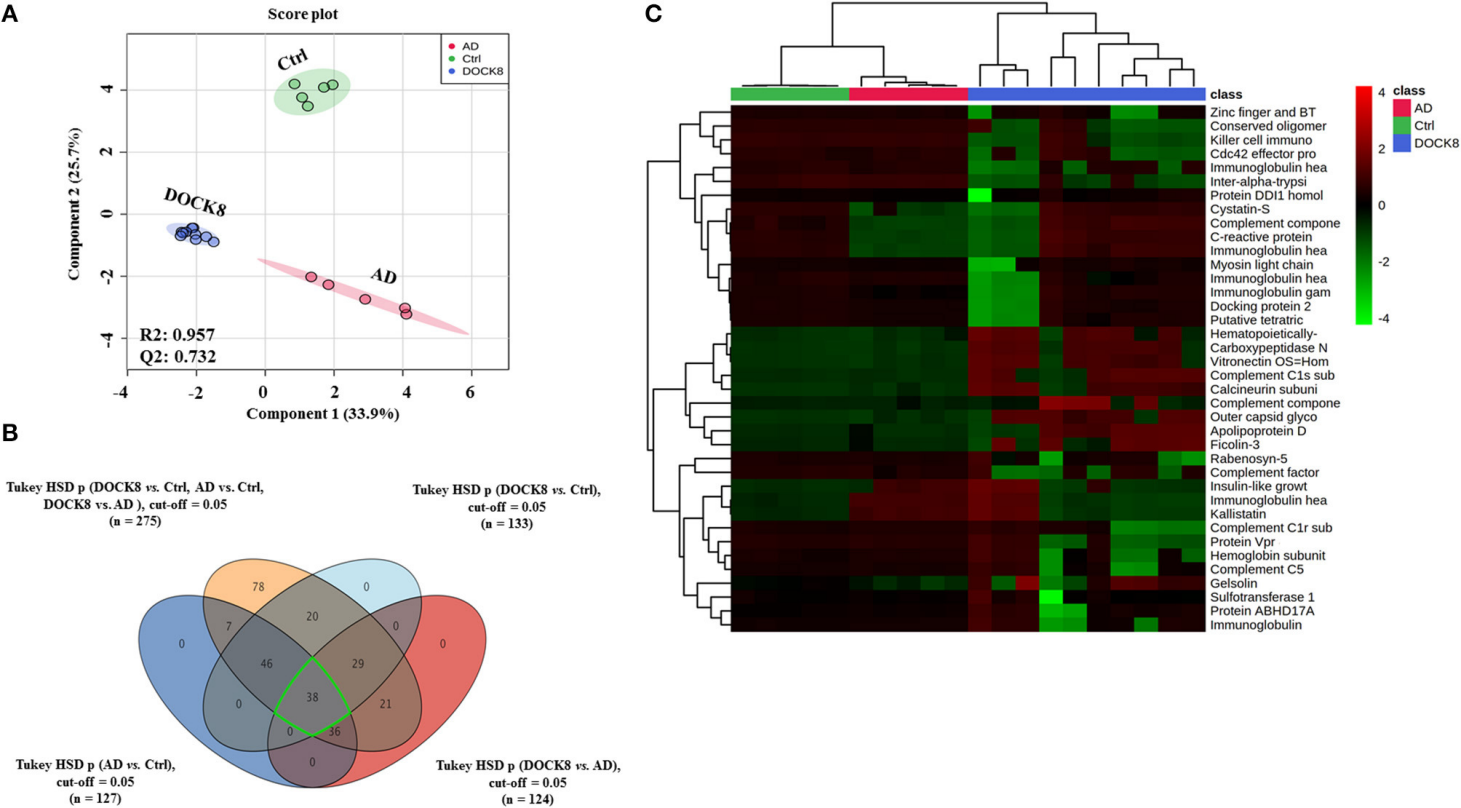
Positively identified proteins in patients with dedicator of cytokinesis 8 (DOCK8) deficiency vs. AD vs. Ctrls. **(A)** PLS-DA score plot between patients with DOCK8 deficiency, patients with AD, and Ctrls (*R*2 = 0.957, *Q*2 = 0.732, *p* < 0.001 for 100 permutations). **(B)** A Venn diagram shows the overlap between the statistically significant proteins in each binary comparison based on one-way ANOVA (Tukey's *post-hoc* FDR *p* < 0.05). Out of 275 differentially regulated proteins, 133 were statistically significant between DOCK8 and Ctrl, 127 between AD and Ctrl, and 124 between DOCK8 and AD. **(C)** A heat map for the 38 commonly differentially regulated proteins among the study binary comparisons.

Untargeted label-free proteomic analysis was carried out in triplicate from pooled serum samples of DOCK8 deficiency, AD, and Ctrl groups. The raw data were normalized to ensure all the data were under Gaussian distribution. The protein expression profiles between the three groups were analyzed using one-way ANOVA (FDR *p* < 0.05), revealing significant differences in a panel of 275 proteins with differential regulation between the three groups. Further supervised multivariate statistical analysis was performed using Partial Least-Squares Discriminant Analysis (PLS-DA) score plots to observe the differences between the three groups. The initial proteomics profile shows a clear group separation and sample clustering (*R*2 = 0.957, *Q*2 = 0.732) between the DOCK8-deficient, AD, and Ctrl groups (Figure 1A). One-way ANOVA and Tukey honest significant difference (HSD) analysis (FDR < 0.05) showed significant differential regulation of proteins in comparison of each group, as illustrated in a Venn diagram (Figure 1B). Group-wise comparison with the controls; DOCK8 and AD showed 133 and 127 proteins were statistically significant. Comparison of DOCK8 vs. AD groups revealed differences in 124 proteins (Figure 1B). An overlap between the different groups was determined by Venn diagram analysis that identified proteins (*n* = 38) in common between the groups having significant differential regulations. The identity and the expression of these proteins are represented in a heatmap (Figure 1C).

**Figure 2:**
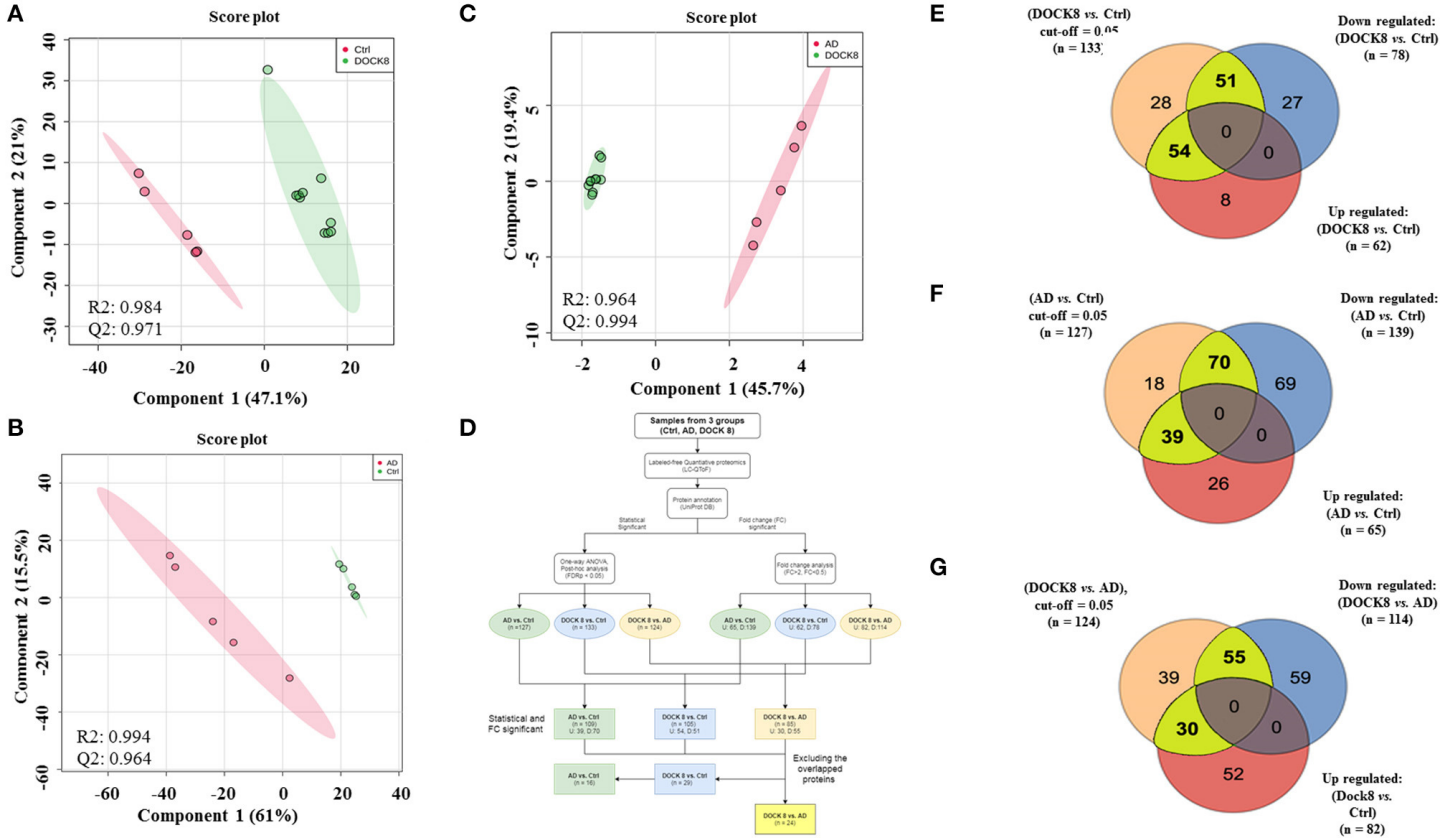
Binary comparison between the study groups using PLS-DA plots, where **(A)** between Ctrl and DOCK8-deficient groups (*R*2 = 0.984 and *Q*2 = 0.0.971), **(B)** AD and Ctrl groups (*R*2 = 0.994 and *Q*2 = 0.0.964), and **(C)** between AD and Ctrl groups (*R*2 = 0.994 and *Q*2 = 0.955). **(D)** A flow chart represents the data analysis workflow. The detected and identified proteins (*n* = 275 proteins) were statistically and fold change analyzed (cut-off FDR *p* < 0.05) and fold change (cut-off 2), respectively. **(E)** 105 significant proteins (statistical and fold change) were found in DOCK8-deficient vs. Ctrl (54 upregulated and 51 downregulated), **(F)** 109 proteins in AD vs. Ctrl (39 upregulated and 70 downregulated), and **(G)** 85 proteins in AD vs. DOCK8-deficient (30 upregulated and 55 downregulated). DOCK8, dedicator of cytokinesis 8; AD, atopic dermatitis; PLS-DA, partial least square discrimination analysis.

The binary comparison was carried out using the PLS-DA score plot between the three groups to ascertain the group separation and clustering of the identified proteins in the data set. A clear separation and clustering were noted between the DOCK8 deficient vs. Ctrls (*R*2 = 0.984 and *Q*2 = 0.971) (Figure 2A) between AD vs. Ctrl groups (*R*2 = 0.994 and *Q*2 = 0.964) (Figure 2B), and DOCK8 deficient vs. AD also showed a clear separation (*R*2 = 0.994 and *Q*2 = 0.955) (Figure 2C). An additional level of analysis using the fold change (FC > 2) was applied to filter out the highly significant proteins from among the identified proteins in the three groups, as described in the flow chart (Figure 2D). From the 133 (FDR *p* < 0.05) differentially regulated proteins in DOCK8 vs. Ctrls, fold change analysis (FC cut-off 2) revealed 105 proteins (54 up and 51 downregulated) to be statistically significant (Supplementary Table 1; Figure 2E). Similarly, from the 127 significantly (FDRp < 0.05) differentially regulated proteins in AD vs. Ctrl groups, fold change analysis (FC cut-off 2) revealed 109 proteins (39 up and 70 downregulated) to be statistically significant (Supplementary Table 2; Figure 2F). Among the 124 significantly (FDR *p* < 0.05) differentially regulated proteins in DOCK8 deficient vs. AD, fold change analysis (FC cut-off 2) revealed 85 proteins (30 up and 55 downregulated) to be statistically significant (Supplementary Table 3; Figure 2G). The clear separation and clustering between the groups indicated that the identified proteins in our data set were specifically altered based on the disease states.

### Biomarker Evaluation

**Figure 3:**
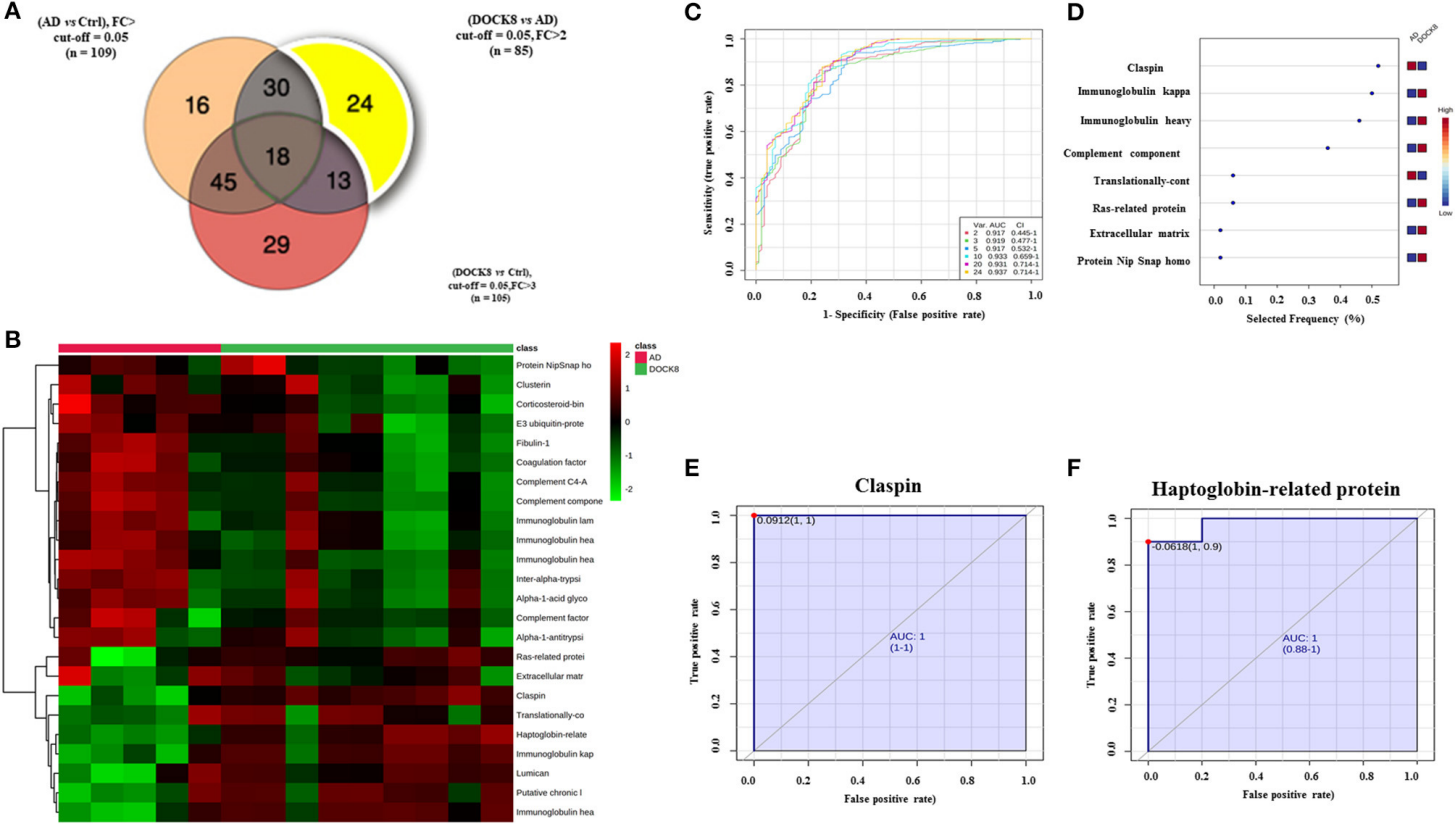
Biomarker evaluation between patients with DOCK8 deficiency and patients with AD based on the proteomics profile. **(A)** A Venn diagram showing 24 proteins differentially uniquely between DOCK8 deficient vs. AD (fold change cut-off; 2, and ANOVA (FDR *p* < 0.05). **(B)** Heat map was created by average reading of the entities hierarchical clustering for the 24 proteins, where the similarity was based on Pearson, which shows significantly differentially regulated proteins specific between DOCK8 and AD groups. **(C)** An exploratory receiver-operating characteristics (ROC) curve generated by the PLS-DA model shows the area under the curve [AUC = 0.937 (0.445-1)] for the top two proteins. **(D)** Frequency plot of differentially expressed proteins between the two groups. **(E)** The ROC curve of Claspin [AUC-1 (1-1)] and **(F)** Haptoglobin-related protein is downregulated in patients with DOCK8 compared to patients with AD [AUC-1 (0.88-1)]. Data were normalized, transformed, and scaled by median, log, and Pareto scaling to ensure all the data were under Gaussian distribution. A combination of ANOVA and fold change analyses is represented for paired analysis, where the x-axis (FDR corrected *p*-value) and the y-axis are true positives. DOCK8, dedicator of cytokines 8; AD, atopic dermatitis.

For the purpose of evaluating potential biomarkers highlighted in the proteomics profile, the significantly differentially regulated proteins (FDR *p*-value < 0.05, FC cut-off 2) among DOCK8 vs. ctrls (*n* = 109), AD vs. Ctrls (*n* = 105), and DOCK8 vs. AD (*n* = 85) were overlapped. Venn diagram analysis highlighted 24 proteins that showed a significant differential regulation, specifically between the DOCK8 deficiency and AD (Figure 3A) after removing the effects of the proteins altered concerning Ctrls. Identity, relative expression, and clustering of these proteins are represented in a heatmap (Figure 3B). The significantly differentially regulated proteins in this panel may serve as potential biomarkers for distinguishing between DOCK8 deficient and AD in clinical practice.

The ROC-exploring curves were generated for these 24 proteins as potential biomarkers for disease detection. The multivariate exploratory ROC analysis was generated using PLS-DA as a classification and feature-ranking method. The combination ROC of the top-ranked metabolites shows the area under the curve (AUC), ranging from 0.917 to 0.937 (Figure 3C; Supplementary Table 4). The sensitivity and specificity of the identified significantly differentially regulated proteins were evaluated individually as potential biomarkers to distinguish between DOCK8 and AD using ROC analysis; the frequency plot of these proteins is represented in Figure 3D using the PLS-DA model, the AUC of claspin (Figure 3E), and haptoglobin-related protein (Hrp) (Figure 3F). ROCs were found as 1, which are strongly recommended biomarkers.

### Proteomics Profile Validation Using Multiple Reaction Monitoring Mass Spectrometry

**Figure 4:**
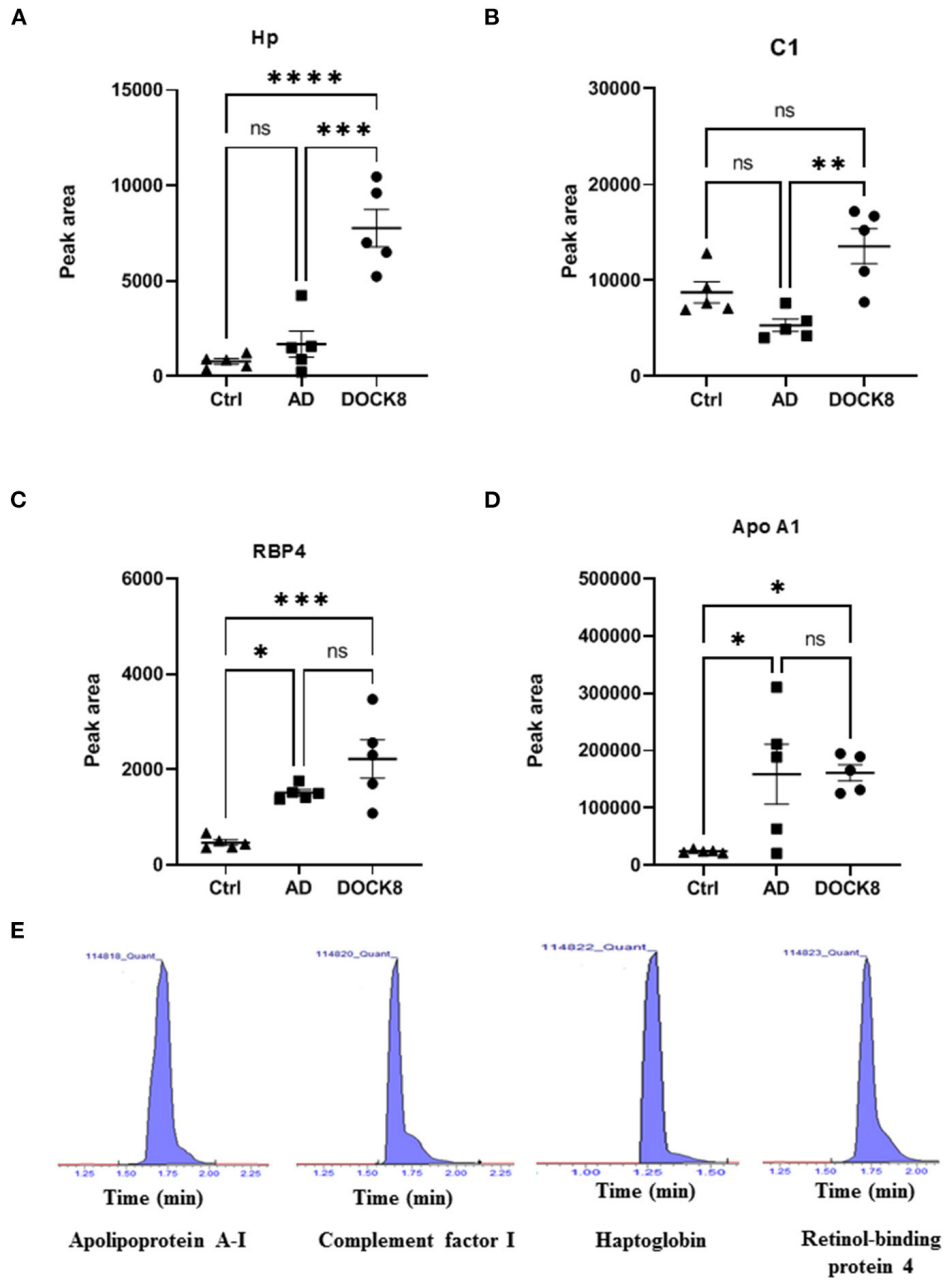
Multiple reaction monitoring (MRM) mass spectrometry for method validation using representative proteins. The MRM method based on signature peptides was developed to validate the expression of four representative proteins **(A)** Hp, Haptoglobin **(B)** C1, complement factor 1 **(C)** RBP4, retinol-binding protein 4, and **(D)** ApoA1, apolipoprotein A-1, found in the proteomics analysis. **(E)** Representative-extracted ion chromatograms for the signature peptides of the four proteins based on the MRM method developed for validation. The expression of these four proteins in patients with DOCK8-deficiency was expressed in the peak area. The statistical significance was evaluated using an unpaired *t*-test, in which ^*^p < 0.05, ^**^p < 0.01, ^***^p < 0.001, and ^****^p < 0.0001.

The signature peptides for the key proteins were selected based on their involvement in the network and pathway analyses as described earlier (17). Four significantly differentially regulated proteins between the proteomics profiles of the study groups were selected for the overall expression validation. Those proteins (Haptoglobin, Complement C1, Retinol-binding protein 4, and Apolipoprotein A1) were interacted and highlighted in multiple networks with significant FC and taken into consideration for further analysis (Figure 4).

The standard materials of signature peptides of proteins were used to develop the multiple reaction monitoring (MRM) transitions by determining the precursor and product ions, collision energy, etc., as detailed in Supplementary Table 5. The patient samples were analyzed using this method to show a concordance with the label-free untargeted proteomics, where representative chromatograms are shown (Figure 4E). Compared to ctrl and AD groups, Hp, C1, and RBP4 were upregulated in patients with DOCK8 deficiency (Figures 4A–C), whereas Apo A1 was upregulated in AD compared to patients with DOCK8 deficiency, and ctrls (Figure 4D).

### Network Pathway Analysis

**Figure 5:**
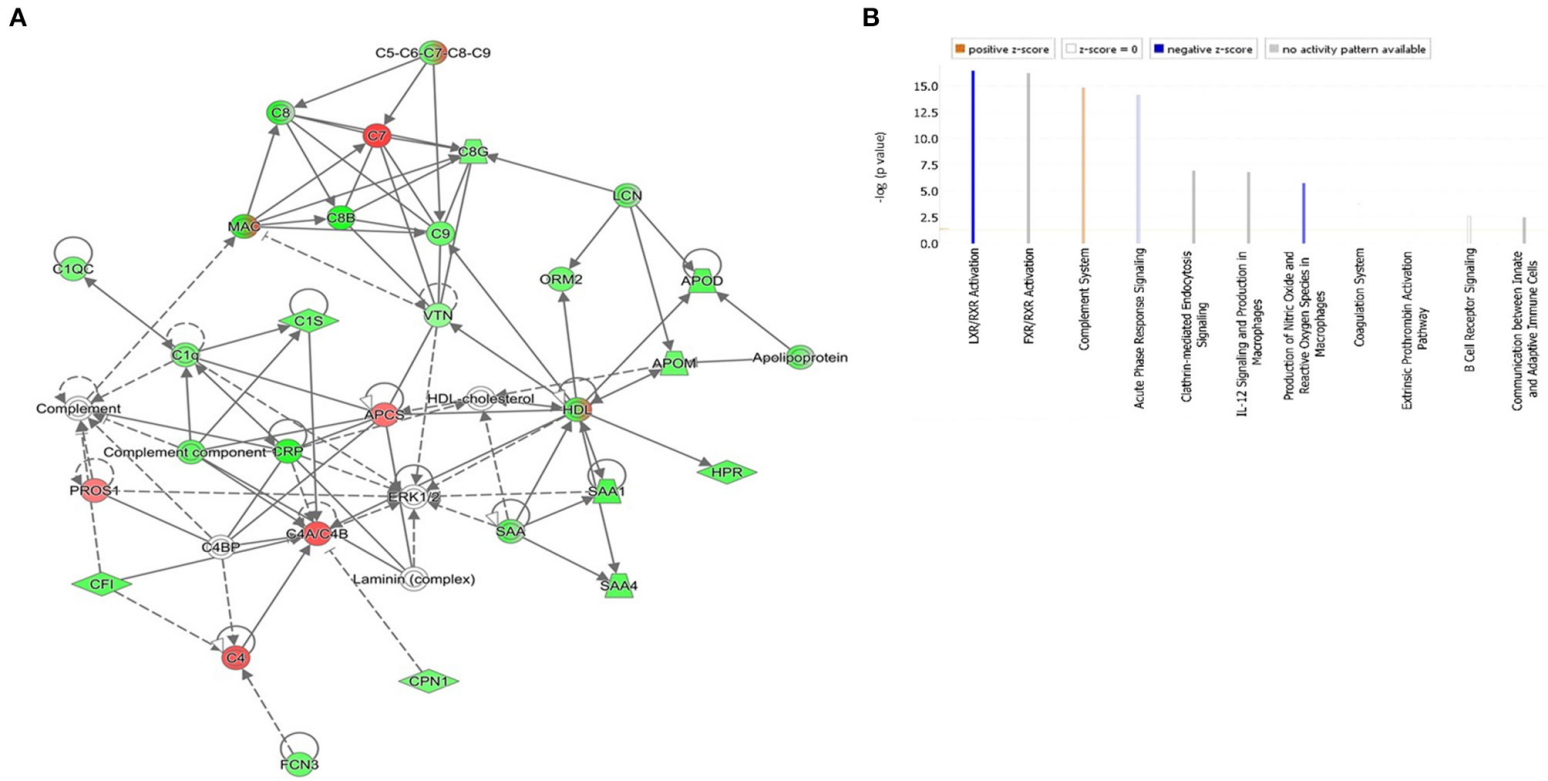
**(A)** The highest-scoring network pathways depict the involvement of the differentially regulated proteins between patients with the highest score of DOCK8 deficiency vs. AD related to the developmental disorder, hereditary disorder, and infectious disease. Nodes in green and red correspond to downregulated and upregulated proteins, respectively. Colorless nodes were proposed by IPA and suggest potential targets functionally coordinated with the differentially abundant proteins. Solid lines indicate direct molecular interactions, and dashed lines represent indirect interactions. **(B)** The significantly enriched top canonical pathways determined by IPA core analysis between DOCK8 deficiency and AD. Activated pathways are colored orange (*Z* > 0), and inhibited pathways are colored blue (*Z* < 0). NA designates pathways without a predicted activity score, which are gray.

The protein-protein interaction and network pathway analysis of the significantly differentially regulated proteins in the three groups were interrogated using the ingenuity pathway analysis (IPA) to identify the involvement of the identified proteins with metabolic pathways. In comparison to the controls, the differentially regulated proteins in DOCK8 deficiency and AD vs. Ctrls both showed involvement of pathways related to humoral immune response, inflammatory response, and cellular maintenance and function (score of 42) with the proteins centered around the extracellular-regulated kinase (ERK ½) and STAT3-signaling pathways in Supplementary Figures 1A, 2A. The IPA revealed the top three canonical pathways in the DOCK8 and AD groups compared to the Ctrl related to acute-phase response signaling, LXR/RXR activation, and FXR/RXR activation (Supplementary Figures 1B, 2B). The comparison of significantly differentially regulated proteins between DOCK8 deficiency vs. AD identified a network pathway related to the developmental disorder, hereditary disorder, and infectious disease with the highest score (score 47). The significantly differentially regulated proteins between DOCK8 deficiency vs. AD centered around the regulation of the ERK1/2-signaling pathway, pointing to its involvement for the common features attributed to both conditions (Figure 5A). The top three canonical pathways enriched by these proteins between DOCK8 deficiency and AD were LXR/RXR activation, FXR/RXR activation, and acute-phase response signaling (Figure 5B).

## Discussion

Dedicator of cytokinesis 8 deficiency is known to have a broad effect on the immune system resulting in combined immunodeficiency (CID), autoimmunity, and atopy with a striking resemblance to severe atopic disease. Although the diagnosis of DOCK8 deficiency, based on the characteristic phenotype, seems straightforward, the presence of mild and atypical phenotypes overlaps with other PIDs and allergic diseases. Moreover, the presentation of dermatitis in patients with DOCK8 deficiency is similar to that typically observed in the classic distribution for AD, making differentiating between these conditions clinically challenging. Currently, the diagnosis of DOCK8 deficiency relies on genetic testing that is arduous and not easily available, resulting in delays in initiating treatment (21).

Proteins are important analytes that change with disease and are amenable to clinical diagnosis. In the present study, we carried out serum proteomic profiling between patients with DOCK8 deficiency and AD using a label-free LC-MS approach. A panel of 24 significantly differentially regulated proteins was identified between the DOCK8 deficiency vs. AD groups. The identified proteins functionally belonged to the class of immunoglobulins (Ig) (IgHV chain, Ig kappa, and Ig lambda light chains), enzymes, complement proteins (C1, C4a, and C7), apolipoproteins, acute-phase proteins (alpha 1 antitrypsin, alpha 1 acid glycoprotein 2, haptoglobin-related protein (Hpr), extracellular matrix proteins (lumican, fibulin, and extracellular matrix protein 1), E3 ubiquitin-protein ligase (a component of UPS), and others.

Defects in DOCK8 are known to lead to severe combined immunodeficiency syndromes, with morphological, developmental, and functional abnormalities of T- and B-cells (2). Compared to the controls, patients with DOCK8 deficiency and AD showed distinct differences between Ig isotype classes. IgG and Ig A levels were upregulated in DOCK8 deficiency compared to AD, while levels of IgM were significantly decreased in DOCK8 deficiency (Figure 1C; Supplementary Tables 1, 2) and were noted (22). The differences in the levels of the immunoglobulins showed that IgG tended to be either normal or elevated, IgA levels were usually within the normal range, while Ig M levels were decreased (9). Decreased IgM levels, known to be the first Ig synthesized by the B cells, may reflect an increase in the class-switching recombinations within B-cells, leading to increased IgG, IgA, and IgE under the influence of altered T-cells and the cytokine profile (11). Mutation of the DOCK8 protein profoundly affects humoral immunity, failing to sustain an antibody response and germinal center B cell persistence. It also reduces activation, survival, proliferation, and Ig secretion by naive B cells in response to stimulation through various surface receptors, including cytokine receptors (23). Besides, the differences in the levels of different immunoglobulin classes were noted in heavy and light chains of our study, which make up individual monomers of the Ig. Abnormalities of B-cells (increase in pre-B-cell subsets, disturbances in memory, and naïve subsets) or presence of a pro-inflammatory state are known to increase levels of by-products of immunoglobulin synthesis, including the constant and variable portions of both heavy and light chains (Supplementary Tables 1, 2; Figure 1C) (24). We found a significant increase in the abundance of the Ig light chains (kappa and lambda) in DOCK8 vs. AD and the IGHV chains (Supplementary Table 3; Figure 3A). Our findings align with Tang et al., who showed a similar skewing of the IGHV chains in patients with DOCK8 deficiency (25). Further studies are needed to determine whether the heavy and light chains result from increased synthesis or breakdown in patients with DOCK8 deficiency.

Claspin, an adaptor, and a scaffold protein were significantly decreased in the DOCK8-deficient group compared to AD. Claspin is a nuclear protein involved in DNA replication, and the DNA damage response is likely to be found at replication forks. It is also involved in regulating the proficiency of the cell cycle, terminating checkpoint-mediated cell cycle arrest (26), maintaining control of DNA replication and the DNA damage response that prevents oncogenic transformation (27). Aberrant increase in expression of claspin has been observed in cancer (28). Besides these functions, activation of signal transducer and activator of transcription 3 (STAT3) was found to promote loss of claspin that facilitated cell proliferation tumorigenesis (29) and increased risk of malignancy as seen in patients with DOCK8 deficiency (Figure 3A; Supplementary Table 3) (6). The association of claspin with STAT3 may support its role as a potential biomarker in identifying and differentiating between DOCK8 deficiency and AD. On further interrogation, claspin had the highest selected frequency (%) and showed the highest discriminatory potential between the two groups (Figure 3E).

We found a decrease in the levels of Hrp, a structural and functional homolog of Hp that acts as acute-phase protein, antioxidant, and high-affinity hemoglobin (Hb)-binding protein. Hrp and Hp binding to Hb prevents its clearance from the plasma by macrophages without breaking down and releasing free heme. Animal studies have shown that free heme interferes with DOCK8-mediated Cdc42 activation, inducing extensive actin cytoskeleton changes and strongly suppresses phagocyte functions predisposing to bacterial dissemination and sepsis (30). A decrease in the heme-scavenging proteins renders patients susceptible to increased bacterial infections, as seen in patients with DOCK8 deficiency, who present with a wide spectrum of infections and lymphopenia with a T-cell senescence profile (27). Hrp was the second protein with the highest discriminatory potential between the two groups (Figure 3F).

Significant differential regulation in the complement pathway components was found in between DOCK8 vs. AD (Supplementary Table 3). Complement Factor 1 was increased in patients with DOCK8 deficiency, while complement Factors 7 and 4a decreased compared to AD. Complement proteins through the classical pathway play an important role in inflammation by bridging between the innate and adaptive immune responses (31). Our proteomic profiling also identified a differential regulation of proteins belonging to the extracellular matrix (ECM) between DOCK8 deficiency vs. AD; lumicanfibulin and extracellular matrix protein 1. The ECM is at the forefront of the immune response. It conveys specific signals to immune cells and allows complex interactions between host tissues to regulate cellular signaling and immune cell dynamics (32). Dysregulation in the organization and deposition of ECMs leads to many pathophysiological conditions exacerbated by aberrant ECM-immune cell interactions. Levels of lumican, a proteoglycan were found to be increased in DOCK8 deficiency compared to AD. Besides regulating collagen fibrillogenesis in connective tissues, it also regulates immune cell recruitment and mediating innate immune responses (33, 34). Fibulin 1 and extracellular matrix protein 1 (ECM1) are secreted glycoproteins associated with cell proliferation, differentiation, ECM formation, and rebuilding. Increased fibulin 1 and ECM1 have been associated with different malignancies (35, 36), and, in cases of breast cancer, it was shown to elicit a TH2 response (37). Further studies are needed to study the role of these proteins in DOCK8 deficiency.

Network pathway analysis of the differentially regulated proteins identified in the data set between DOCK8 deficiency and AD centered around the ERK1/2 signaling pathway with the highest connectivity. Pathways enriched by the identified proteins provide an insight into the potential biochemical mechanisms utilized by the proteins in the different disease states. The ERK1/2 pathway regulates cell proliferation, migration, differentiation, and cytokine/mediator secretion *via* activation of transcription factors (38). The ERK-signaling pathways are critical to developing and differentiating the innate and adaptive immune response cells, including the T cells (39). A recent proteomic study identified the role of ERK signaling in the control of CD8+ differentiation and in T cell communication with other immune cells for mediating adaptive immune responses. While, in the B cell, ERK signaling is important for antigen receptor-induced proliferation and development (40, 41). In a recent study, ERK1/2 signaling was found to play a role in TH2 cell differentiation and the allergic response (42, 43). The dysregulation of the ERK1/2 signaling pathway by the significantly differentially regulated proteins points to its central role in immune dysregulation between both DOCK8 deficiency vs. AD (Figure 5A).

Our present study has extended our previous work that showed alterations in the cytokine and metabolite profile and has now extended it by demonstrating distinct alterations in the proteome between DOCK8 deficiency and AD. Our study has several limitations. Although we carefully matched the patient profiles between DOCK8 deficiency, we could not control the significant eosinophilia seen in patients with DOCK8 deficiency; moreover, the changes of the proteins between DOCK8 deficiency and AD did not allude to any proteins responsible for T lymphocytes or alterations in the cytokine profile.

Our study is not without limitations. The samples in our cohort had a skewed gender ratio with a larger number of females in the AD group. Moreover, obtaining the healthy control pediatric samples for the study was challenging. This limited us in providing the demographic data for the healthy control group as the de-identified age and sex matched samples were obtained from a biobank of samples collected from healthy subjects during their regular checkups in pediatric family clinic.

In summary, the plasma proteomic profiling of DOCK8 deficiency revealed the complex nature of the disease with differential regulation of multiple proteins, regulating various metabolic pathways. Besides, the profiling of DOCK8 deficiency and AD groups revealed a shared role of ERK1/2 through the commonly differentially regulated proteins in the disease states that may contribute to similar clinical features.

## Data Availability Statement

The original contributions presented in the study are publicly available. The mass spectrometry proteomics data has been deposited to the ProteomeXchange Consortium *via* the PRIDE partner repository, with the dataset identifier PXD029052.

## Ethics Statement

The studies involving human participants were reviewed and approved by Research Ethics Committee at the Office of Research Affairs of King Faisal Specialist Hospital and Research Centre (KFSHRC) (RAC No. 2160015). Written informed consent to participate in this study was provided by the participants' legal guardian/next of kin.

## Author Contributions

MJ collected the samples, conducted the experiments, built the figures, performed data analysis, and drafted the manuscript. AM performed integrated data analysis and contributed to drafting the manuscript. RA, BA, and HA-M recruited patients and provided their clinical and genetic data and samples. AAA conducted the proteomics analysis and reviewed the manuscript. ZS and MA aided with the proteomics analysis. MD critically revised the manuscript. AMA designed the study, supervised experiments, data analysis, and finalized the manuscript. All authors approved the final version of the manuscript.

## Conflict of Interest

The authors declare that the research was conducted in the absence of any commercial or financial relationships that could be construed as a potential conflict of interest.

## Publisher's Note

All claims expressed in this article are solely those of the authors and do not necessarily represent those of their affiliated organizations, or those of the publisher, the editors and the reviewers. Any product that may be evaluated in this article, or claim that may be made by its manufacturer, is not guaranteed or endorsed by the publisher.
