# Supplementary material for: Proteomics Profiling to Distinguish DOCK8 Deficiency From Atopic Dermatitis
Source: Front Allergy. 2021 Nov 29;2:774902. doi: 10.3389/falgy.2021.774902 (PMC8974780; doi:10.3389/falgy.2021.774902)
Supplement: Supplementary file 1 [file Table_1.docx]

**Table S1:** List of 54 up and 51 down regulated proteins in DOCK8 compared to Ctrls with fold change analysis >2. (G105)

| **Uniprot Accession number** | **Protein names** | **Log Fold change (FC)** | **P-value** |
| --- | --- | --- | --- |
| Q6YN16 | Hydroxysteroid dehydrogenase-like protein 2 | 1.99 | 0.0002 |
| Q8WTU0 | Protein DDI1 homolog 1 | 1.83 | 0.0002 |
| Q02045 | Myosin light chain 5 | 1.80 | 0.0002 |
| Q73369 | Protein Vpr | 0.82 | 0.0002 |
| O75897 | Sulfotransferase 1C4 | 1.40 | 0.0002 |
| Q96GS6 | Protein ABHD17A | 0.83 | 0.0210 |
| O60496 | Docking protein 2 | 1.92 | 0.0002 |
| Q6P2S7;P55036; Q13490;Q6P0N0;Q7L099;Q96LZ2 | Putative tetratricopeptide repeat protein 41 | 1.97 | 0.0002 |
| P0DOX5;P01857 | Immunoglobulin gamma-1 heavy chain | 2.30 | 0.0002 |
| P10381 | Protease | 1.59 | 0.0050 |
| P19827 | Inter-alpha-trypsin inhibitor heavy chain H1 | -1.19 | 0.0205 |
| P01877 | Immunoglobulin heavy constant alpha 2 | 1.32 | 0.0011 |
| Q9Y2V7 | Conserved oligomeric Golgi complex subunit 6 | 1.04 | 0.0006 |
| Q9H8W3 | Protein FAM204A | 1.99 | 0.0002 |
| P15169 | Carboxypeptidase N catalytic chain | -1.08 | 0.0014 |
| P09871 | Complement C1s subcomponent | -0.98 | 0.0006 |
| P11856;P10501 | Outer capsid glycoprotein VP7 | -1.11 | 0.0012 |
| P01871 | Immunoglobulin heavy constant mu | -1.45 | 0.0002 |
| P0DJI8 | Serum amyloid A-1 protein | 1.98 | 0.0002 |
| P01036 | Cystatin-S | 2.06 | 0.0002 |
| P02765 | Alpha-2-HS-glycoprotein | -1.74 | 0.0002 |
| P01764;P01768 | Immunoglobulin heavy variable 3-23 | -1.75 | 0.0008 |
| P01861 | Immunoglobulin heavy constant gamma 4 | 2.05 | 0.0002 |
| Q9H1K0 | Rabenosyn-5 | 1.33 | 0.0002 |
| P02760 | Protein AMBP | -2.07 | 0.0002 |
| A6NDY0 | Embryonic polyadenylate-binding protein 2 | 2.19 | 0.0002 |
| P02750 | Leucine-rich alpha-2-glycoprotein | 1.10 | 0.0329 |
| P29466 | Caspase-1 | -1.50 | 0.0009 |
| O95365 | Zinc finger and BTB domain-containing protein 7A | 1.30 | 0.0002 |
| P69891;P69892 | Hemoglobin subunit gamma-1 | 0.76 | 0.0281 |
| P04544 | Non-structural protein 1 | -1.85 | 0.0002 |
| P0DJI9 | Serum amyloid A-2 protein | 2.23 | 0.0002 |
| Q9NZP8 | Complement C1r subcomponent-like protein | 0.70 | 0.0150 |
| Q14954 | Killer cell immunoglobulin-like receptor 2DS1 | 1.09 | 0.0050 |
| O00291 | Huntingtin-interacting protein 1 | 2.20 | 0.0002 |
| Q9UH36 | SRR1-like protein | -1.81 | 0.0002 |
| P01031;O95711; Q8TAG9 | Complement C5 | 1.08 | 0.0002 |
| P69905;P02008 | Hemoglobin subunit alpha | -1.62 | 0.0035 |
| Q86T23 | Putative ciliary rootlet coiled-coil protein-like 1 protein | -1.89 | 0.0002 |
| P02741 | C-reactive protein | 1.02 | 0.0047 |
| Q09328 | Alpha-1_6-mannosylglycoprotein 6-beta-N-acetylglucosaminyltransferase A | -1.76 | 0.0003 |
| P22792 | Carboxypeptidase N subunit 2 | -2.06 | 0.0002 |
| P01762 | Immunoglobulin heavy variable 3-11 | 1.63 | 0.0002 |
| P20851 | C4b-binding protein beta chain | -1.95 | 0.0002 |
| Q9UPW5 | Cytosolic carboxypeptidase 1 | -1.83 | 0.0004 |
| P52460 | Putative CC-type chemokine U83 | -1.90 | 0.0002 |
| P35858 | Insulin-like growth factor-binding protein complex acid labile subunit | -2.06 | 0.0002 |
| P05090 | Apolipoprotein D | -0.77 | 0.0117 |
| O60938 | Keratocan | -1.46 | 0.0070 |
| P06396 | Gelsolin | 1.78 | 0.0002 |
| P04220 | Ig mu heavy chain disease protein | -1.25 | 0.0008 |
| P0DOX7 | Immunoglobulin kappa light chain | 1.82 | 0.0002 |
| Q92973 | Transportin-1 | -1.90 | 0.0002 |
| P52438 | Inner tegument protein | -1.53 | 0.0027 |
| P08697 | Alpha-2-antiplasmin | -1.98 | 0.0002 |
| A8MYB1 | Transmembrane and coiled-coil domain-containing protein 5B | 1.84 | 0.0004 |
| P02042 | Hemoglobin subunit delta | -1.54 | 0.0007 |
| Q9BV99 | Leucine-rich repeat-containing protein 61 | 1.18 | 0.0152 |
| P01743;A0A0C4DH29 | Immunoglobulin heavy variable 1-46 | 1.52 | 0.0002 |
| P07225 | Vitamin K-dependent protein S | -1.70 | 0.0010 |
| P01876 | Immunoglobulin heavy constant alpha 1 | 1.35 | 0.0002 |
| P18428 | Lipopolysaccharide-binding protein | 1.70 | 0.0004 |
| P07358 | Complement component C8 beta chain | 1.08 | 0.0003 |
| Q9NQ66 | 1-phosphatidylinositol 4_5-bisphosphate phosphodiesterase beta-1 | -1.82 | 0.0005 |
| P43652 | Afamin | -1.83 | 0.0002 |
| P63098 | Calcineurin subunit B type 1 | -1.57 | 0.0002 |
| P02655 | Apolipoprotein C-II | -1.69 | 0.0011 |
| Q06033 | Inter-alpha-trypsin inhibitor heavy chain H3 | 1.05 | 0.0115 |
| Q9Y2B9 | cAMP-dependent protein kinase inhibitor gamma | 1.62 | 0.0038 |
| P01834 | Immunoglobulin kappa constant | 2.15 | 0.0002 |
| Q9H596 | Dual specificity protein phosphatase 21 | -1.80 | 0.0002 |
| P02652 | Apolipoprotein A-II | -1.85 | 0.0003 |
| Q15573 | TATA box-binding protein-associated factor RNA polymerase I subunit A | -1.29 | 0.0278 |
| P80748 | Immunoglobulin lambda variable 3-21 | 1.35 | 0.0277 |
| P36980 | Complement factor H-related protein 2 | 1.44 | 0.0003 |
| Q9H3Q1 | Cdc42 effector protein 4 | 1.29 | 0.0037 |
| Q03701 | CCAAT/enhancer-binding protein zeta | -1.93 | 0.0002 |
| P01023;Q16880 | Alpha-2-macroglobulin | -1.71 | 0.0004 |
| P01023;Q16880 | Alpha-1-antichymotrypsin | 2.05 | 0.0002 |
| Q13434;Q9UHC7 | Putative E3 ubiquitin-protein ligase makorin-4 | -1.72 | 0.0012 |
| Q8WXX5 | DnaJ homolog subfamily C member 9 | -1.39 | 0.0021 |
| Q9UBS0 | Ribosomal protein S6 kinase beta-2 | -1.66 | 0.0027 |
| P04196 | Histidine-rich glycoprotein | -1.78 | 0.0007 |
| P19823 | Inter-alpha-trypsin inhibitor heavy chain H2 | -1.61 | 0.0040 |
| P08603;Q02985 | Complement factor H | 1.64 | 0.0032 |
| O75037 | Kinesin-like protein KIF21B | -1.92 | 0.0002 |
| P02100 | Hemoglobin subunit epsilon | -1.37 | 0.0249 |
| P01591 | Immunoglobulin J chain | 1.13 | 0.0090 |
| P01591 | Uncharacterized protein C8orf48 | -1.86 | 0.0004 |
| P50771 | Regulatory protein E2 | 1.79 | 0.0003 |
| P02766 | Transthyretin | -1.51 | 0.0097 |
| P06727 | Apolipoprotein A-IV | -2.00 | 0.0002 |
| O14791 | Apolipoprotein L1 | -1.27 | 0.0141 |
| P02647 | Apolipoprotein A-I | -1.47 | 0.0048 |
| Q8IWJ2 | GRIP and coiled-coil domain-containing protein 2 | -1.50 | 0.0101 |
| P02787;O95267;P50502;P52823;Q8IZP2;Q8NFI4 | Serotransferrin | -1.55 | 0.0005 |
| A0A0A0MS15 | Immunoglobulin heavy variable 3-49 | 1.65 | 0.0022 |
| Q92797 | Symplekin | 1.95 | 0.0002 |
| P42336 | Phosphatidylinositol 4_5-bisphosphate 3-kinase catalytic subunit alpha isoform | -1.87 | 0.0002 |
| P36385 | Processing and transport protein (Fragment) | 1.71 | 0.0016 |
| P02743 | Serum amyloid P-component | -1.56 | 0.0032 |
| P02654 | Apolipoprotein C-I | -2.01 | 0.0002 |
| A0A0B4J2D9 | Immunoglobulin kappa variable 1D-13 | -1.45 | 0.0122 |
| P78358 | Cancer/testis antigen 1 | 1.26 | 0.0150 |
| Q14192 | Four and a half LIM domains protein 2 | 1.38 | 0.0149 |
