# Supplementary material for: Proteomics Profiling to Distinguish DOCK8 Deficiency From Atopic Dermatitis
Source: Front Allergy. 2021 Nov 29;2:774902. doi: 10.3389/falgy.2021.774902 (PMC8974780; doi:10.3389/falgy.2021.774902)
Supplement: Supplementary file 2 [file Table_2.docx]

**Table S2**: List of 70 up and 39 down regulated proteins in AD compared to Ctrls with fold change analysis >2. (G109)

| **Uniprot Accession number** | **Protein names** | **Log Fold change (FC)** | **P-value** |
| --- | --- | --- | --- |
| Q8WTU0 | Protein DDI1 homolog 1 | 2.6 | 0.0002 |
| Q02045 | Myosin light chain 5 | 2.6 | 0.0002 |
| Q73369 | Protein Vpr | 2.58 | 0.0002 |
| O75897 | Sulfotransferase 1C4 | 2.53 | 0.0002 |
| Q96GS6 | Protein ABHD17A | 2.37 | 0.0002 |
| O60496 | Docking protein 2 | 2.55 | 0.0002 |
| Q6P2S7;P55036;Q13490;Q6P0N0;Q7L099;Q96LZ2 | Putative tetratricopeptide repeat protein 41 | 2.56 | 0.0002 |
| P0DOX5;P01857 | Immunoglobulin gamma-1 heavy chain | 2.01 | 0.0002 |
| P19827 | Inter-alpha-trypsin inhibitor heavy chain H1 | -2 | 0.0012 |
| P02671 | Fibrinogen alpha chain | -1.51 | 0.0323 |
| Q9Y2V7 | Conserved oligomeric Golgi complex subunit 6 | 2.53 | 0.0002 |
| Q9H8W3 | Protein FAM204A | 1.47 | 0.0025 |
| P15169 | Carboxypeptidase N catalytic chain | -2.47 | 0.0002 |
| P09871 | Complement C1s subcomponent | -2.55 | 0.0002 |
| P11856;P10501 | Outer capsid glycoprotein VP7 | -2.47 | 0.0002 |
| P01036 | Cystatin-S | 0.97 | 0.018 |
| P02765 | Alpha-2-HS-glycoprotein | -2.18 | 0.0002 |
| P01764;P01768 | Immunoglobulin heavy variable 3-23 | -1.55 | 0.007 |
| P01861 | Immunoglobulin heavy constant gamma 4 | 1.78 | 0.0002 |
| Q9H1K0 | Rabenosyn-5 | 2.67 | 0.0002 |
| P02760 | Protein AMBP | -2.12 | 0.0002 |
| A6NDY0 | Embryonic polyadenylate-binding protein 2 | 1.86 | 0.0002 |
| P01859 | Immunoglobulin heavy constant gamma 2 | -2 | 0.0005 |
| Q14568 | Heat shock protein HSP 90-alpha A2 | -2.29 | 0.0002 |
| O95365 | Zinc finger and BTB domain-containing protein 7A | 2.63 | 0.0002 |
| P69891;P69892 | Hemoglobin subunit gamma-1 | 2.37 | 0.0002 |
| P04544 | Non-structural protein 1 | -2.14 | 0.0002 |
| P0DJI9 | Serum amyloid A-2 protein | 1.95 | 0.0002 |
| Q9NZP8 | Complement C1r subcomponent-like protein | 2.44 | 0.0002 |
| Q9H7L9 | Sin3 histone deacetylase corepressor complex component SDS3 | -1.25 | 0.0119 |
| P02748 | Complement component C9 | -1.41 | 0.034 |
| Q14954 | Killer cell immunoglobulin-like receptor 2DS1 | 2.37 | 0.0002 |
| P01782 | Immunoglobulin heavy variable 3-9 | -1.62 | 0.0195 |
| O00291 | Huntingtin-interacting protein 1 | 2.14 | 0.0002 |
| Q9UH36 | SRR1-like protein | -1.96 | 0.0003 |
| P01031;O95711; Q8TAG9 | Complement C5 | 2.64 | 0.0002 |
| Q03591 | Complement factor H-related protein 1 | 1.53 | 0.014 |
| P69905;P02008 | Hemoglobin subunit alpha | -1.36 | 0.031 |
| Q86T23 | Putative ciliary rootlet coiled-coil protein-like 1 protein | -1.71 | 0.0011 |
| P02741 | C-reactive protein | -1.06 | 0.0099 |
| O00750 | Phosphatidylinositol 4-phosphate 3-kinase C2 domain-containing subunit beta | 2.07 | 0.0007 |
| Q96LS8 | Uncharacterized protein C2orf48 | -1.58 | 0.0003 |
| P22792 | Carboxypeptidase N subunit 2 | -1.72 | 0.0003 |
| P01762 | Immunoglobulin heavy variable 3-11 | 2.57 | 0.0002 |
| Q9UPW5 | Cytosolic carboxypeptidase 1 | -1.63 | 0.0028 |
| P35858 | Insulin-like growth factor-binding protein complex acid labile subunit | -1.08 | 0.0109 |
|  | Apolipoprotein D | -2.44 | 0.0002 |
| P05543 | Thyroxine-binding globulin | -1.71 | 0.0038 |
| P06396 | Gelsolin | 2.54 | 0.0002 |
| O95445 | Apolipoprotein M | -1.9 | 0.0017 |
| P0DOX7 | Immunoglobulin kappa light chain | 2.24 | 0.0002 |
| Q92973 | Transportin-1 | -1.95 | 0.0002 |
| P08697 | Alpha-2-antiplasmin | -1.33 | 0.0066 |
| P01602 | Immunoglobulin kappa variable 1-5 | 1.62 | 0.0209 |
| P0DOX4 | Immunoglobulin epsilon heavy chain | -1.28 | 0.0269 |
| O75636 | Ficolin-3 | -2.35 | 0.0002 |
| A8MYB1 | Transmembrane and coiled-coil domain-containing protein 5B | 1.13 | 0.0411 |
| P04278 | Sex hormone-binding globulin | -1.39 | 0.0438 |
| O14727 | Apoptotic protease-activating factor 1 | -1.4 | 0.0246 |
| Q9BV99 | Leucine-rich repeat-containing protein 61 | 2.09 | 0.0006 |
| P01743;A0A0C4DH29 | Immunoglobulin heavy variable 1-46 | 2.58 | 0.0002 |
| P07360 | Complement component C8 gamma chain | -2.46 | 0.0002 |
| P01876 | Immunoglobulin heavy constant alpha 1 | -0.77 | 0.0129 |
| P00738 | Haptoglobin | -1.41 | 0.0071 |
| Q9UIF8;Q8N1M1 | Bromodomain adjacent to zinc finger domain protein 2B | 2.05 | 0.0008 |
| A0A087WW87; P01614 | Immunoglobulin kappa variable 2-40 | 1.56 | 0.0004 |
| P07358 | Complement component C8 beta chain | -1.12 | 0.0006 |
| Q9NQ66 | 1-phosphatidylinositol 4_5-bisphosphate phosphodiesterase beta-1 | -1.47 | 0.0083 |
| P63098 | Calcineurin subunit B type 1 | -2.39 | 0.0002 |
| P02655 | Apolipoprotein C-II | -1.74 | 0.0027 |
| Q06033 | Inter-alpha-trypsin inhibitor heavy chain H3 | 2.3 | 0.0002 |
| P35542;Q05111 | Serum amyloid A-4 protein | -2.15 | 0.0002 |
| P02649 | Apolipoprotein E | -1.93 | 0.0003 |
| P29622 | Kallistatin | 0.74 | 0.0252 |
| Q9H596 | Dual specificity protein phosphatase 21 | -2.15 | 0.0002 |
| Q15573 | TATA box-binding protein-associated factor RNA polymerase I subunit A | -1.57 | 0.02 |
| P04217 | Alpha-1B-glycoprotein | -2.11 | 0.0004 |
| Q03014 | Hematopoietically-expressed homeobox protein HHEX | -2.11 | 0.0005 |
| Q03701 | CCAAT/enhancer-binding protein zeta | -1.08 | 0.0321 |
| P01023;Q16880 | Alpha-2-macroglobulin | -1.98 | 0.0004 |
| P0CF74 | Immunoglobulin lambda constant 6 | -1.7 | 0.0003 |
| P25311 | Zinc-alpha-2-glycoprotein | -1.47 | 0.0116 |
| P04196 | Histidine-rich glycoprotein | -1.45 | 0.0108 |
| O75037 | Kinesin-like protein KIF21B | -1.13 | 0.027 |
| P16499 | Rod cGMP-specific 3'_5'-cyclic phosphodiesterase subunit alpha | -1.81 | 0.0031 |
| Q96LL4 | Uncharacterized protein C8orf48 | -1.34 | 0.0136 |
| P02753 | Retinol-binding protein 4 | -1.35 | 0.012 |
| P01854 | Immunoglobulin heavy constant epsilon | -1.61 | 0.0088 |
| B0I1T2 | Unconventional myosin-Ig | -1.56 | 0.0264 |
| P04004 | Vitronectin | -2.42 | 0.0002 |
| P06727 | Apolipoprotein A-IV | -2.1 | 0.0002 |
| P40855 | Peroxisomal biogenesis factor 19 | -1.87 | 0.0034 |
| O14791 | Apolipoprotein L1 | -1.96 | 0.0015 |
| P02775 | Platelet basic protein | -1.43 | 0.037 |
| Q9UHR6 | Zinc finger HIT domain-containing protein 2 | -1.47 | 0.0277 |
| P0C7P4 | Putative cytochrome b-c1 complex subunit Rieske-like protein 1 | -1.78 | 0.0036 |
| P36955 | Pigment epithelium-derived factor | -1.7 | 0.0115 |
| P00747; Q02325 | Plasminogen | -1.77 | 0.0024 |
| Q92797 | Symplekin | 1.49 | 0.0033 |
| P02747 | Complement C1q subcomponent subunit C | -1.45 | 0.0338 |
| P03952 | Plasma kallikrein | -2.03 | 0.001 |
| P02654 | Apolipoprotein C-I | -1.83 | 0.0002 |
| P78358 | Cancer/testis antigen 1 | 1.97 | 0.0015 |
| Q5JSJ4; Q5TBE3 | Integrator complex subunit 6-like | -1.36 | 0.0363 |
| P02788 | Lactotransferrin | 2.08 | 0.0004 |
| P02774 | Vitamin D-binding protein | -2.1 | 0.0003 |
| P02776;P10720 | Platelet factor 4 | -1.42 | 0.0333 |
| Q96PD5 | N-acetylmuramoyl-L-alanine amidase | 1.28 | 0.0386 |
| P05452 | Tetranectin | 1.27 | 0.0283 |
