# Supplementary material for: Proteomics Profiling to Distinguish DOCK8 Deficiency From Atopic Dermatitis
Source: Front Allergy. 2021 Nov 29;2:774902. doi: 10.3389/falgy.2021.774902 (PMC8974780; doi:10.3389/falgy.2021.774902)
Supplement: Supplementary file 3 [file Table_3.docx]

**Table S3**: List of 30 up and 55 down regulated proteins in AD compared to DOCK8 with fold change analysis >2. (G85)

| **Uniprot Accession number** | **Protein names** | **Log Fold change (FC)** | **P-value** |
| --- | --- | --- | --- |
| Q6YN16 | Hydroxysteroid dehydrogenase-like protein 2 | -1.56 | 0.0002 |
| Q8WTU0 | Protein DDI1 homolog 1 | 0.77 | 0.0002 |
| Q73369 | Protein Vpr | 1.76 | 0.0002 |
| O75897 | Sulfotransferase 1C4 | 1.13 | 0.0004 |
| Q96GS6 | Protein ABHD17A | 1.54 | 0.0002 |
| P0DOX5; P01857 | Immunoglobulin gamma-1 heavy chain | -0.29 | 0.021 |
| P01877 | Immunoglobulin heavy constant alpha 2 | -1.92 | 0.0002 |
| P15169 | Carboxypeptidase N catalytic chain | -1.39 | 0.0002 |
| P09871 | Complement C1s subcomponent | -1.56 | 0.0002 |
| P11856; P10501 | Outer capsid glycoprotein VP7 | -1.37 | 0.0003 |
| P0DJI8 | Serum amyloid A-1 protein | -1.77 | 0.0002 |
| P01036 | Cystatin-S | -1.09 | 0.0027 |
| Q9H1K0 | Rabenosyn-5 | 1.34 | 0.0002 |
| P01859 | Immunoglobulin heavy constant gamma 2 | -1.62 | 0.0009 |
| Q14568 | Heat shock protein HSP 90-alpha A2 | -1.91 | 0.0002 |
| Q9HAW4 | Claspin | -1.8 | 0.0005 |
| P02750 | Leucine-rich alpha-2-glycoprotein | -1.65 | 0.0018 |
| P29466 | Caspase-1 | 1.7 | 0.0003 |
| Q9H7L9 | Sin3 histone deacetylase corepressor complex component SDS3 | -1.96 | 0.0002 |
| P02748 | Complement component C9 | -1.48 | 0.0103 |
| P01031;O95711;Q8TAG9 | Complement C5 | 1.56 | 0.0002 |
| P02741 | C-reactive protein | -2.08 | 0.0002 |
| Q96LS8 | Uncharacterized protein C2orf48 | -2.14 | 0.0002 |
| P52460 | Putative CC-type chemokine U83 | 0.94 | 0.0337 |
| P35858 | Insulin-like growth factor-binding protein complex acid labile subunit | 0.99 | 0.0073 |
| P05090 | Apolipoprotein D | -1.67 | 0.0002 |
| P05543 | Thyroxine-binding globulin | -1.66 | 0.0015 |
| P23083 | Immunoglobulin heavy variable 1-2 | 1.64 | 0.0023 |
| P00739 | Haptoglobin-related protein | -1.65 | 0.0027 |
| O95445 | Apolipoprotein M | -1.48 | 0.0041 |
| P0DOX4 | Immunoglobulin epsilon heavy chain | -1.78 | 0.0008 |
| O75636 | Ficolin-3 | -1.5 | 0.0003 |
| P05156 | Complement factor I | -1.62 | 0.0036 |
| P04278 | Sex hormone-binding globulin | -1.37 | 0.0219 |
| O14727 | Apoptotic protease-activating factor 1 | -1.62 | 0.0033 |
| P02042 | Hemoglobin subunit delta | 1.68 | 0.0003 |
| P01743;A0A0C4DH29 | Immunoglobulin heavy variable 1-46 | 1.06 | 0.0003 |
| P07225 | Vitamin K-dependent protein S | 1.16 | 0.0177 |
| P07360 | Complement component C8 gamma chain | -1.4 | 0.0002 |
| P01876 | Immunoglobulin heavy constant alpha 1 | -2.12 | 0.0002 |
| P00738 | Haptoglobin | -1.9 | 0.0003 |
| A0A087WW87;P01614 | Immunoglobulin kappa variable 2-40 | 2.11 | 0.0002 |
| P07358 | Complement component C8 beta chain | -2.21 | 0.0002 |
| P0DOX8;B9A064;P0CG04 | Immunoglobulin lambda-1 light chain | -1.82 | 0.0004 |
| P63098 | Calcineurin subunit B type 1 | -0.82 | 0.0185 |
| P35542; Q05111 | Serum amyloid A-4 protein | -1.64 | 0.0004 |
| P04217 | Alpha-1B-glycoprotein | -1.32 | 0.0056 |
| Q9H3Q1 | Cdc42 effector protein 4 | 0.95 | 0.0304 |
| Q03014 | Hematopoietically-expressed homeobox protein HHEX | -1.04 | 0.0332 |
| P23142 | Fibulin-1 | 1.51 | 0.001 |
| P0CF74 | Immunoglobulin lambda constant 6 | -2.08 | 0.0002 |
| P25311 | Zinc-alpha-2-glycoprotein | -1.75 | 0.001 |
| Q9UFN0 | Protein NipSnap homolog 3A | 1.62 | 0.0039 |
| P16499 | Rod cGMP-specific 3'_5'-cyclic phosphodiesterase subunit alpha | -1.5 | 0.0043 |
| P01591 | Immunoglobulin J chain | -1.88 | 0.0002 |
| P01854 | Immunoglobulin heavy constant epsilon | -1.59 | 0.0032 |
| P04004 | Vitronectin | -1.07 | 0.0031 |
| P50771 | Regulatory protein E2 | -1.28 | 0.0044 |
| P40855 | Peroxisomal biogenesis factor 19 | -1.22 | 0.0241 |
| Q5K130 | Putative chronic lymphocytic leukemia up-regulated protein 1 opposite strand transcript protein | -1.62 | 0.004 |
| P00748 | Coagulation factor XII | 1.44 | 0.013 |
| P01009 | Alpha-1-antitrypsin | -1.79 | 0.0007 |
| P20338 | Ras-related protein Rab-4A | -1.45 | 0.0144 |
| P51884 | Lumican | -1.31 | 0.0335 |
| A0A0C4DH55;P01624 | Immunoglobulin kappa variable 3D-7 | -1.58 | 0.0039 |
| P02775 | Platelet basic protein | -1.37 | 0.0203 |
| Q9UHR6 | Zinc finger HIT domain-containing protein 2 | -1.43 | 0.0134 |
| P00747;Q02325 | Plasminogen | -1.67 | 0.0012 |
| P0C0L4 | Complement C4-A | 1.47 | 0.0114 |
| A0A0C4DH41;P01824;P01825;P06331 | Immunoglobulin heavy variable 4-61 | -1.56 | 0.0059 |
| P02747 | Complement C1q subcomponent subunit C | -1.36 | 0.0213 |
| P42336 | Phosphatidylinositol 4_5-bisphosphate 3-kinase catalytic subunit alpha isoform | 1.51 | 0.0003 |
| P10643 | Complement component C7 | 1.65 | 0.0029 |
| P02743 | Serum amyloid P-component | 1.24 | 0.017 |
| Q14624 | Inter-alpha-trypsin inhibitor heavy chain H4 | -1.37 | 0.0263 |
| P08185 | Corticosteroid-binding globulin | 1.18 | 0.0109 |
| P13693 | Translationally-controlled tumor protein | 1.22 | 0.0351 |
| P02788 | Lactotransferrin | 1.51 | 0.0015 |
| P10909 | Clusterin | -1.77 | 0.0009 |
| P19652;Q7L1V2;P54707 | Alpha-1-acid glycoprotein 2 | -1.49 | 0.0114 |
| P01772 | Immunoglobulin heavy variable 3-33 | 1.37 | 0.017 |
| Q8IWV7 | E3 ubiquitin-protein ligase UBR1 | 1.27 | 0.0309 |
| Q16610 | Extracellular matrix protein 1 | 1.45 | 0.009 |
| Q96PD5 | N-acetylmuramoyl-L-alanine amidase | 1.68 | 0.0021 |
| P05452 | Tetranectin | 1.78 | 0.0008 |
