# Supplementary material for: Proteomics Profiling to Distinguish DOCK8 Deficiency From Atopic Dermatitis
Source: Front Allergy. 2021 Nov 29;2:774902. doi: 10.3389/falgy.2021.774902 (PMC8974780; doi:10.3389/falgy.2021.774902)
Supplement: Supplementary file 4 [file Table_4.docx]

**Table S4:** List of 24 proteins (G24) with ROCs and P-values.

| **Protein names** | **AUC** | **P-value** |
| --- | --- | --- |
| Claspin | 1 | 7.24E-05 |
| Haptoglobin-related protein | 0.97778 | 4.17E-04 |
| Corticosteroid-binding globulin | 0.97778 | 0.0036223 |
| Immunoglobulin heavy variable 1-2 | 0.95556 | 7.33E-04 |
| Immunoglobulin kappa variable 3D-7 | 0.95556 | 0.0024877 |
| Immunoglobulin heavy variable 4-61 | 0.93333 | 0.0017847 |
| Fibulin-1 | 0.91111 | 0.0064864 |
| Complement component C7 | 0.88889 | 0.0045415 |
| Immunoglobulin heavy variable 3-33 | 0.88889 | 0.021586 |
| Coagulation factor XII | 0.86667 | 0.014772 |
| Complement C4-A | 0.84444 | 0.01132 |
| E3 ubiquitin-protein ligase UBR1 | 0.82222 | 0.040405 |
| Immunoglobulin lambda-1 light chain | 0.8 | 0.078281 |
| Putative chronic lymphocytic leukemia up-regulated protein 1 opposite strand transcript protein | 0.77778 | 0.021235 |
| Ras-related protein Rab-4A | 0.77778 | 0.045036 |
| Inter-alpha-trypsin inhibitor heavy chain H4 | 0.77778 | 0.037643 |
| Clusterin | 0.75556 | 0.14044 |
| Alpha-1-acid glycoprotein 2 | 0.75556 | 0.0923 |
| Lumican | 0.73333 | 0.033532 |
| Protein NipSnap homolog 3A | 0.68889 | 0.5484 |
| Translationally-controlled tumor protein | 0.66667 | 0.19374 |
| Alpha-1-antitrypsin | 0.66667 | 0.23189 |
| Complement factor 1 | 0.64444 | 0.31024 |
| Extracellular matrix protein 1 | 0.51111 | 0.88184 |
