# Supplementary material for: Proteomics Profiling to Distinguish DOCK8 Deficiency From Atopic Dermatitis
Source: Front Allergy. 2021 Nov 29;2:774902. doi: 10.3389/falgy.2021.774902 (PMC8974780; doi:10.3389/falgy.2021.774902)
Supplement: Supplementary file 5 [file Table_5.docx]

| **Accession Number** | **Protein Name** | **Peptide Sequence [location]** | **Length (AA)** | **Alignment score BLAST/NCBI** | **Molecular weight (g/mol)** | **MRM Transition** | | **Cone voltage (v)** | **Collision energy (v)** | **RT (min)** |
| --- | --- | --- | --- | --- | --- | --- | --- | --- | --- | --- |
|  | | | | | | **MS1 (m/z)** | **MS2 (m/z)** |  | | |
| P02647 (D) | Apolipoprotein A-I (APOA1) | R.EQLGPVTQEFWDNLEK.E [85, 100] | 16 | 50-80 | 1933.2 | 967.67 | 84.08 | 100 | 98 | 1.75 |
| P05156 (D) | Complement factor I (C3b/C4b) | R.VFSLQWGEVK.L [480, 489] | 10 | <40 | 1192.3 | 597 | 120.07 | 38 | 40 | 1.73 |
| P00738 (D) | Haptoglobin (Hp) | R.TEGDGVYTLNNEK.Q [118, 130] | 13 | 40-50 | 1439.5 | 720.58 | 84.15 | 44 | 78 | 1.26 |
| P02753 (D) | Retinol-binding protein 4 (RBP4) | K.YWGVASFLQK.G [107, 116] | 10 | <40 | 1198.4 | 600.22 | 136.11 | 36 | 56 | 1.76 |

**Table S5:** List of proteins used for validation based on signature peptides in MRM method
