# Supplementary material for: Proteomics Profiling to Distinguish DOCK8 Deficiency From Atopic Dermatitis
Source: Front Allergy. 2021 Nov 29;2:774902. doi: 10.3389/falgy.2021.774902 (PMC8974780; doi:10.3389/falgy.2021.774902)
Supplement: Supplementary file 6 [file Presentation_1.PPTX]

## Slide 1
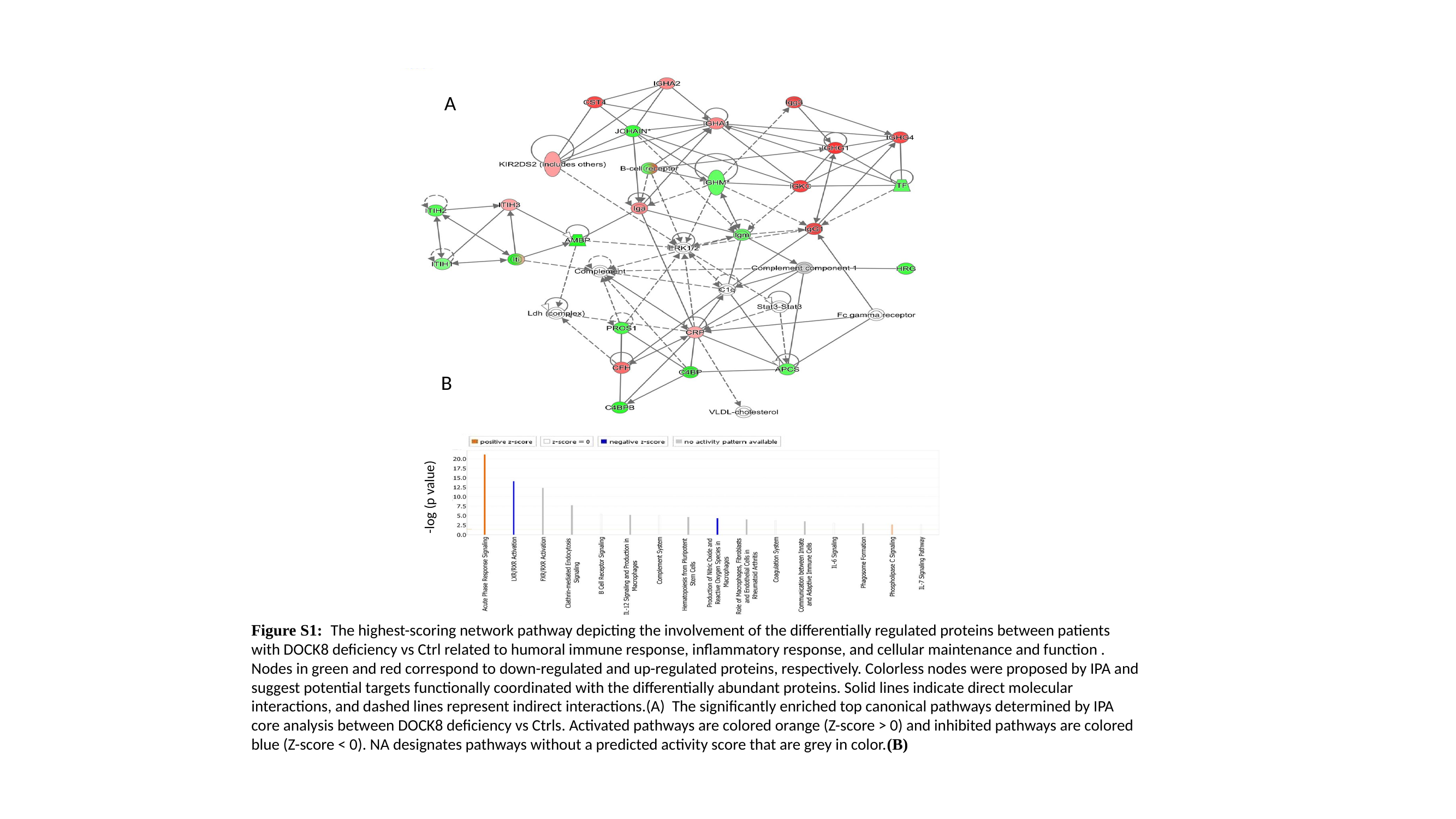

-log (p value)
A
B
Figure S1: The highest-scoring network pathway depicting the involvement of the differentially regulated proteins between patients with DOCK8 deficiency vs Ctrl related to humoral immune response, inflammatory response, and cellular maintenance and function . Nodes in green and red correspond to down-regulated and up-regulated proteins, respectively. Colorless nodes were proposed by IPA and suggest potential targets functionally coordinated with the differentially abundant proteins. Solid lines indicate direct molecular interactions, and dashed lines represent indirect interactions.(A) The significantly enriched top canonical pathways determined by IPA core analysis between DOCK8 deficiency vs Ctrls. Activated pathways are colored orange (Z-score > 0) and inhibited pathways are colored blue (Z-score < 0). NA designates pathways without a predicted activity score that are grey in color.(B)

## Slide 2
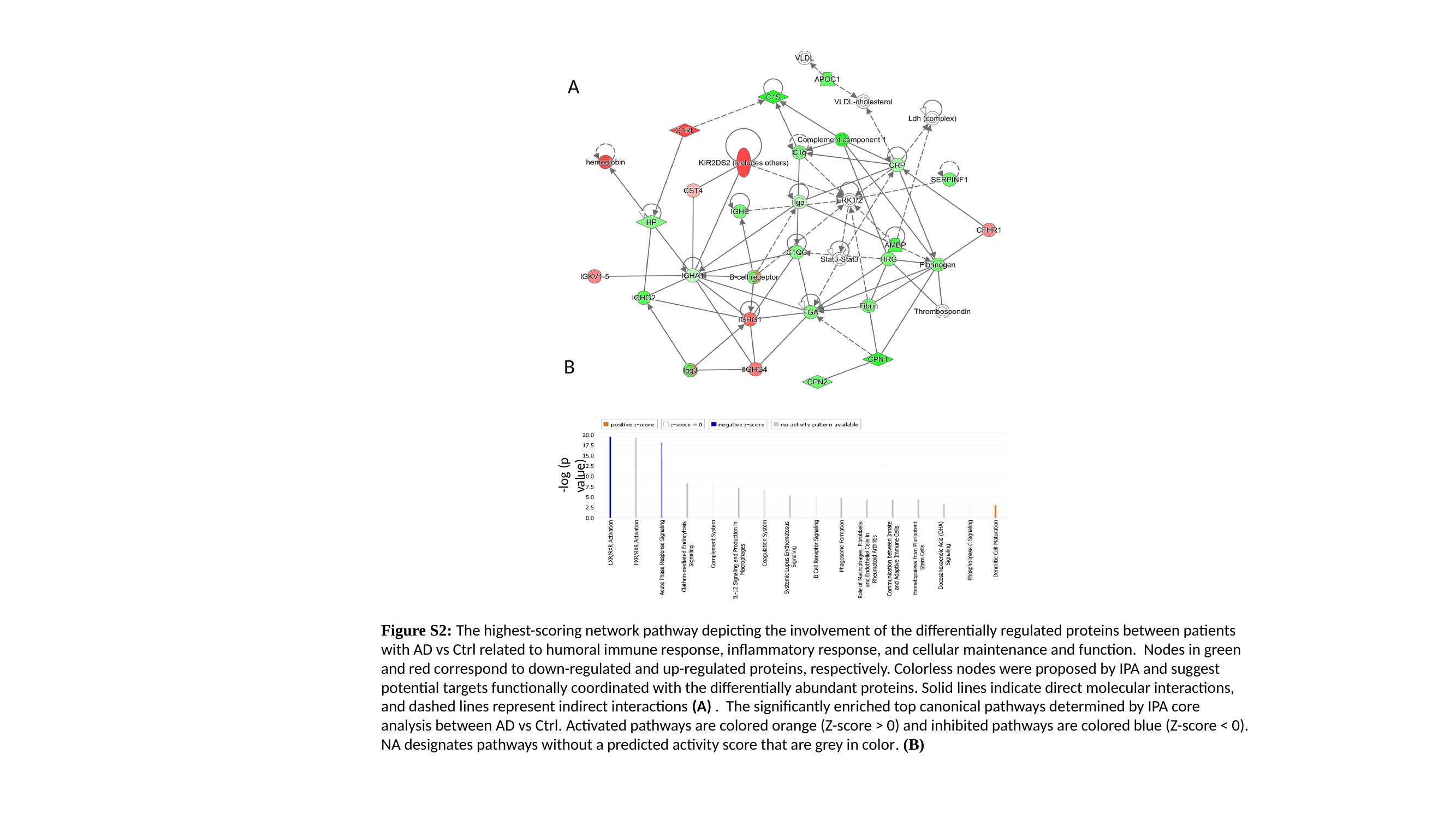

A
B
-log (p value)
Figure S2: The highest-scoring network pathway depicting the involvement of the differentially regulated proteins between patients with AD vs Ctrl related to humoral immune response, inflammatory response, and cellular maintenance and function. Nodes in green and red correspond to down-regulated and up-regulated proteins, respectively. Colorless nodes were proposed by IPA and suggest potential targets functionally coordinated with the differentially abundant proteins. Solid lines indicate direct molecular interactions, and dashed lines represent indirect interactions (A) . The significantly enriched top canonical pathways determined by IPA core analysis between AD vs Ctrl. Activated pathways are colored orange (Z-score > 0) and inhibited pathways are colored blue (Z-score < 0). NA designates pathways without a predicted activity score that are grey in color. (B)

## Slide 3
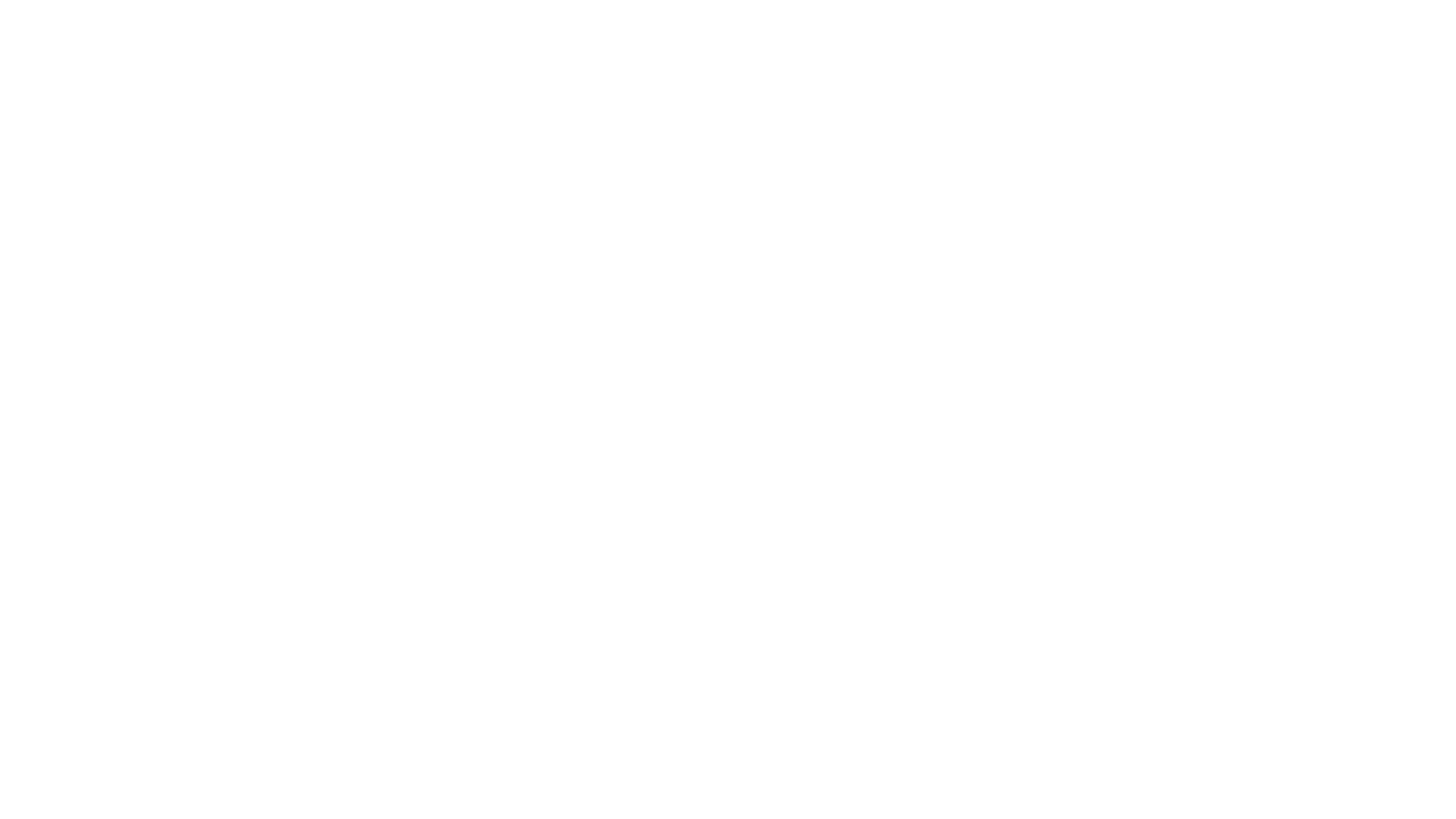

## Slide 4
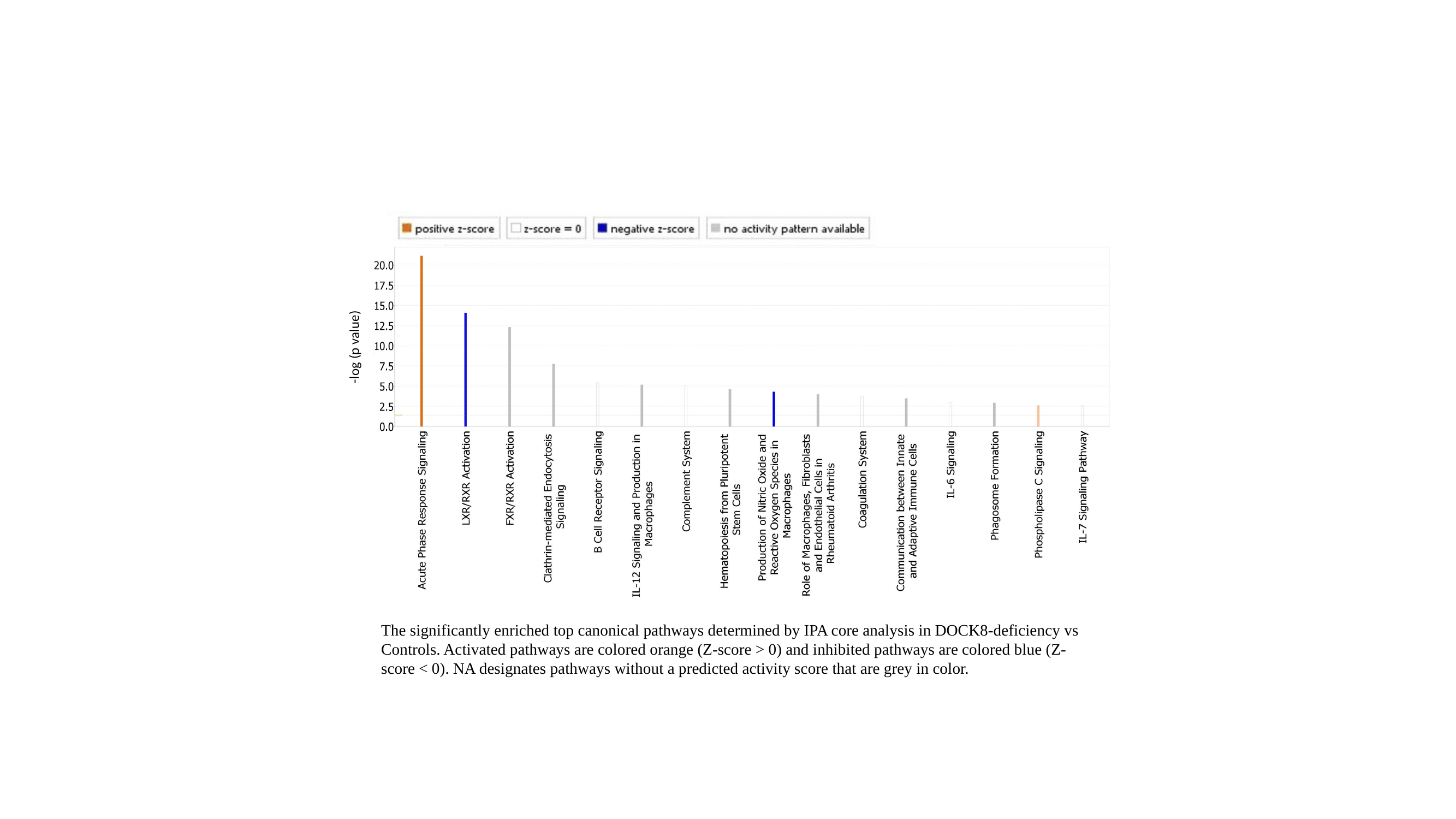

-log (p value)
The significantly enriched top canonical pathways determined by IPA core analysis in DOCK8-deficiency vs Controls. Activated pathways are colored orange (Z-score > 0) and inhibited pathways are colored blue (Z-score < 0). NA designates pathways without a predicted activity score that are grey in color.

## Slide 5
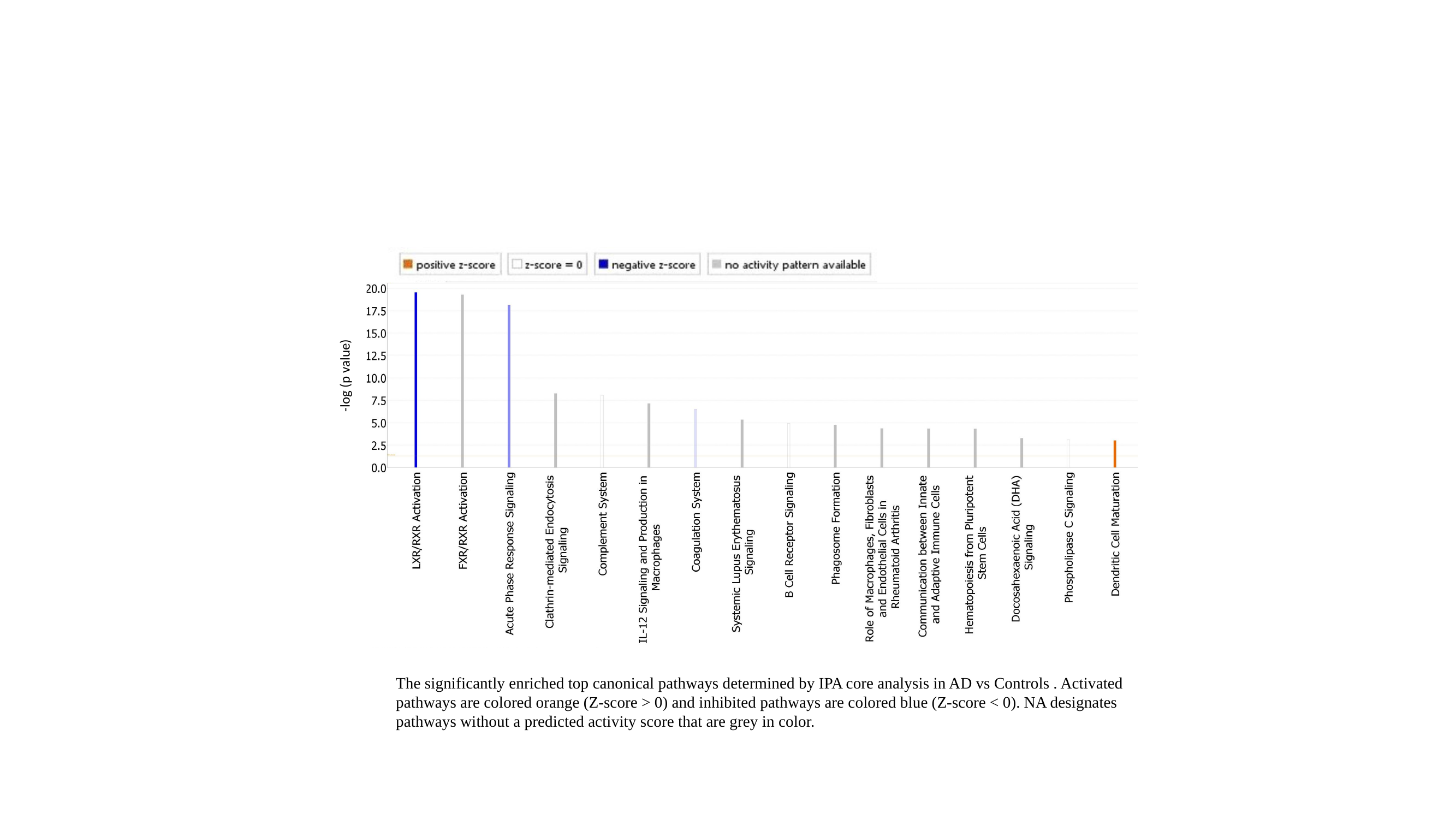

-log (p value)
The significantly enriched top canonical pathways determined by IPA core analysis in AD vs Controls . Activated pathways are colored orange (Z-score > 0) and inhibited pathways are colored blue (Z-score < 0). NA designates pathways without a predicted activity score that are grey in color.

## Slide 6
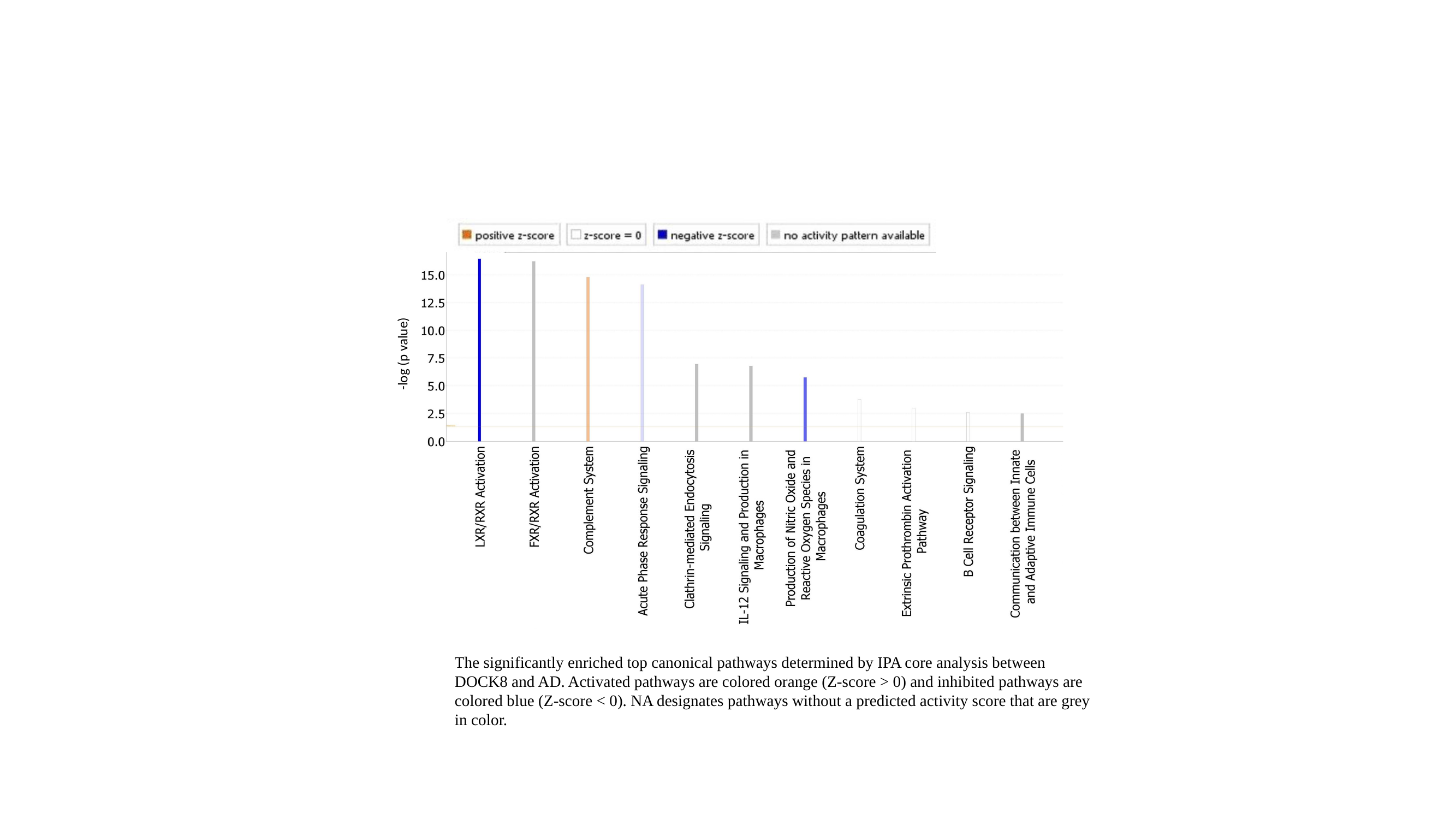

-log (p value)
The significantly enriched top canonical pathways determined by IPA core analysis between DOCK8 and AD. Activated pathways are colored orange (Z-score > 0) and inhibited pathways are colored blue (Z-score < 0). NA designates pathways without a predicted activity score that are grey in color.
